# Preclinical investigations using [^177^Lu]Lu-Ibu-DAB-PSMA toward its clinical translation for radioligand therapy of prostate cancer

**DOI:** 10.1007/s00259-022-05837-2

**Published:** 2022-05-30

**Authors:** Viviane J. Tschan, Francesca Borgna, Sarah D. Busslinger, Martina Stirn, Josep M. Monné Rodriguez, Peter Bernhardt, Roger Schibli, Cristina Müller

**Affiliations:** 1 Center for Radiopharmaceutical Sciences ETH-PSI-USZ, Paul Scherrer Institute, 5232 Villigen-PSI, Switzerland; 2 Clinical Laboratory, Department of Clinical Diagnostics and Services, Vetsuisse Faculty, University of Zurich, 8057 Zurich, Switzerland; 3 Laboratory for Animal Model Pathology (LAMP), Institute of Veterinary Pathology, Vetsuisse Faculty, University of Zurich, 8057 Zurich, Switzerland; 4 Department of Radiation Physics, Institution of Clinical Science, Sahlgrenska Academy, University of Gothenburg, 41345 Gothenburg, Sweden; 5 Department of Chemistry and Applied Biosciences, ETH Zurich, 8093 Zurich, Switzerland

**Keywords:** PSMA, Prostate cancer, Radioligand therapy, Albumin binder, Lutetium-177

## Abstract

[^177^Lu]Lu-Ibu-DAB-PSMA was previously characterized with moderate albumin-binding properties enabling high tumor accumulation but reasonably low retention in the blood. The aim of this study was to investigate [^177^Lu]Lu-Ibu-DAB-PSMA in preclinical in vivo experiments and compare its therapeutic efficacy and potential undesired side effects with those of [^177^Lu]Lu-PSMA-617 and the previously developed [^177^Lu]Lu-PSMA-ALB-56. BALB/c nude mice without tumors were investigated on Day 10 and 28 after injection of 10 MBq radioligand. It was revealed that most plasma parameters were in the same range for all groups of mice and histopathological examinations of healthy tissue did not show any alternations in treated mice as compared to untreated controls. Based on these results, a therapy study over twelve weeks was conducted with PC-3 PIP tumor-bearing mice for comparison of the radioligands’s therapeutic efficacy up to an activity of 10 MBq (1 nmol) per mouse. In agreement with the increased mean absorbed tumor dose, [^177^Lu]Lu-Ibu-DAB-PSMA (~ 6.6 Gy/MBq) was more effective to inhibit tumor growth than [^177^Lu]Lu-PSMA-617 (~ 4.5 Gy/MBq) and only moderately less potent than [^177^Lu]Lu-PSMA-ALB-56 (~ 8.1 Gy/MBq). As a result, the survival of mice treated with 2 MBq of an albumin-binding radioligand was significantly increased (*p* < 0.05) compared to that of mice injected with [^177^Lu]Lu-PSMA-617 or untreated controls. The majority of mice treated with 5 MBq or 10 MBq [^177^Lu]Lu-Ibu-DAB-PSMA or [^177^Lu]Lu-PSMA-ALB-56 were still alive at study end. Hemograms of immunocompetent mice injected with 30 MBq [^177^Lu]Lu-Ibu-DAB-PSMA or 30 MBq [^177^Lu]Lu-PSMA-617 showed values in the same range as untreated controls. This was, however, not the case for mice treated with [^177^Lu]Lu-PSMA-ALB-56 which revealed a drop in lymphocytes and hemoglobin at Day 10 and Day 28 after injection. The data of this study demonstrated a significant therapeutic advantage of [^177^Lu]Lu-Ibu-DAB-PSMA over [^177^Lu]Lu-PSMA-617 and a more favorable safety profile as compared to that of [^177^Lu]Lu-PSMA-ALB-56. Based on these results, [^177^Lu]Lu-Ibu-DAB-PSMA may has the potential for a clinical translation.

**Supplementary Information:**

The online version contains supplementary material available at 10.1007/s00259-022-05837-2.

## Introduction

Prostate cancer is the worldwide second most frequent cause of cancer-related death in men [1]. Effective treatment of advanced disease remains a major challenge [2]; however, radioligand therapy (RLT) using prostate-specific membrane antigen (PSMA)-targeting radioligands emerged as an effective means for the treatment of patients with metastatic castration-resistant prostate cancer (mCRPC) [3–6]. The Phase III clinical trial (VISION; NCT0351166 [7]) revealed a significantly prolonged progression-free survival (8.7 vs. 3.4 months) and improved overall survival (15.3 vs 11.3 months) of patients treated with [^177^Lu]Lu-PSMA-617 in addition to standard of care compared to standard of care alone [8]. In March 2022, [^177^Lu]Lu-PSMA-617 was approved by the US Food and Drug Administration (FDA) under the trade name Pluvicto for the treatment of adult patients with PSMA-positive mCRPC, who have been treated with androgen receptor pathway inhibition and taxane-based chemotherapy.

In order to further improve the therapeutic outcome of RLT, many research projects focused on chemical modifications of PSMA radioligands to optimize their pharmacokinetics. Several attempts focused on the derivatization of radioligands with 4-(*p*-iodophenyl)butanoate [9] or Evans blue as strong albumin binders to enhance the radioligands’ blood circulation time and, hence, achieve increased tumor accumulation [10–15]. Our group discovered that slightly reduced albumin-binding affinity was more favorable and developed a radioligand with a 4-(*p-*tolyl)butanoate entity [9] conjugated to the PSMA ligand backbone via a lysine residue to obtain [^177^Lu]Lu-PSMA-ALB-56 [16]. Kuo et al*.* came to the same conclusion and used *p-*chlorophenyl and *p-*methoxyphenyl entities as moderate albumin binders for the design of novel PSMA radioligands with improved pharmacokinetic profiles [17]. A recently performed first-in-human clinical application of [^177^Lu]Lu-PSMA-ALB-56 showed, however, still high blood activity levels and kidney retention, which would limit the number of therapy cycles that could be safely applied [16, 18].

**Fig. 1:**
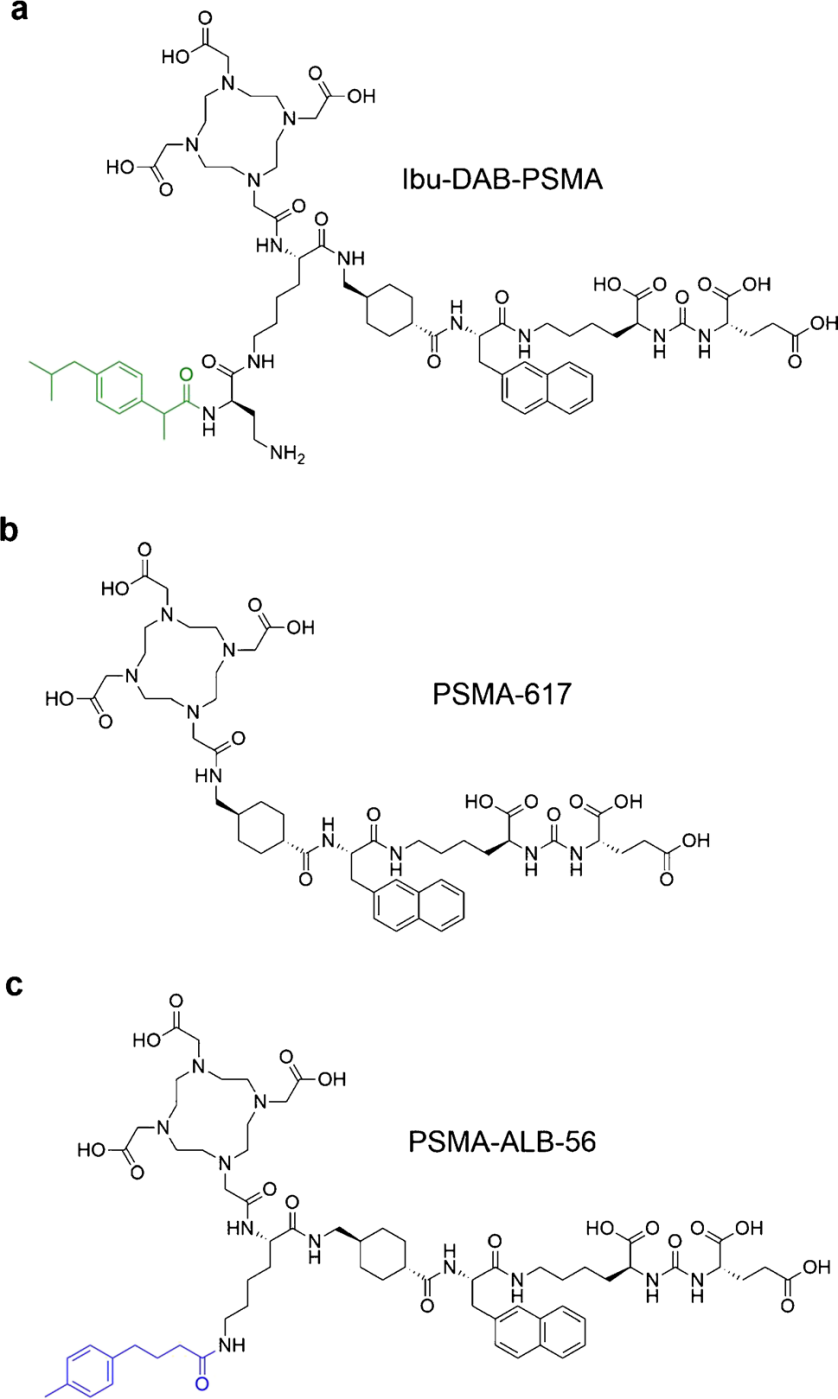
a-c PSMA ligands composed of a glutamine-urea-lysine PSMA-binding entity, a linker entity and a DOTA chelator for coordination of lutetium-177. **a** Ibu-DAB-PSMA modified with ibuprofen (Ibu) (green) as an albumin-binding entity conjugated via a linker entity composed of a diaminobutyric acid (DAB) and a lysine residue [19]; **b** PSMA-617 without dedicated albumin binder [20]; **c** PSMA-ALB-56 modified with a 4-(*p*-tolyl)butanoate entity (blue) as an albumin binding entity conjugated via lysine residue [16]

In an attempt to achieve higher tumor uptake than observed with [^177^Lu]Lu-PSMA-617, but faster clearance from the blood as compared to that of [^177^Lu]Lu-PSMA-ALB-56, a series of novel PSMA ligands were developed with ibuprofen as an albumin-binding entity conjugated via variable linker entities [19]. [^177^Lu]Lu-Ibu-DAB-PSMA (Fig. 1), the most promising candidate, had similar in vitro characteristics as [^177^Lu]Lu-PSMA-617 in terms of stability, PSMA-binding affinity as well as cell uptake in PSMA-expressing tumor cells [19]. Importantly, the area under the curve of non-decay-corrected biodistribution data (AUC_0→192 h_), showed a 1.4-fold higher tumor uptake for [^177^Lu]Lu-Ibu-DAB-PSMA as compared to [^177^Lu]Lu-PSMA-617 and a 2.6-fold lower blood retention than for [^177^Lu]Lu-PSMA-ALB-56 [11, 16, 19]. These differences could be ascribed to the moderate binding of [^177^Lu]Lu-Ibu-DAB-PSMA to blood plasma proteins, which appeared favorable to achieve the desired pharmacokinetic profile.

The aim of this study was to investigate [^177^Lu]Lu-Ibu-DAB-PSMA in preclinical settings to evaluate the therapeutic efficacy and tolerability in mice and compare the data with those of [^177^Lu]Lu-PSMA-617 and [^177^Lu]Lu-PSMA-ALB-56.

## Materials and methods

### Estimated mean absorbed dose to tumors and kidneys

Dosimetry calculations were performed based on non-decay-corrected biodistribution data obtained from previously performed studies with PC-3 PIP tumor-bearing mice (Supplementary Material) [11, 16, 19]. The cumulated activity was estimated by calculating the time-integrated activity concentration coefficients (TIACCs) and used for calculation of the mean specific absorbed dose (Gy/MBq) to the tumor xenografts and to the kidneys. The absorbed fractions for the tumors and the kidneys were assessed by Monte Carlo simulations using PENELOPE (Supplementary Material) [21].

### Radiolabeling of PSMA ligands

The radiolabeling of the PSMA ligands with lutetium-177 (no-carrier-added; in 0.04 M HCl; ITM Medical Isotopes GmbH, Germany) was performed under standard labeling conditions at pH 4.5 as previously reported [16]. The radioligands were obtained with ≥ 98% radiochemical purity at a molar activity of up to 30 MBq/nmol and used without additional purification steps (Supplementary Material, Fig. S1).

### Cell culture

PSMA-positive PC-3 PIP cells, a subline of the androgen-independent PC-3 human prostate cancer cell line, transduced to express PSMA [22, 23], were kindly provided by Prof. Dr. Martin Pomper (Johns Hopkins University School of Medicine, Baltimore, MD, U.S.A.). The tumor cells were cultured under standard culturing conditions using RPMI-1640 cell culture medium supplemented with 10% fetal calf serum, antibiotics and puromycin (2 µg/mL) [16].

### In vivo studies

All applicable international, national, and/or institutional guidelines for the care and use of animals were followed, and the experiments were performed according to the guidelines of the Swiss regulations for animal welfare. The studies were ethically approved by the cantonal committee of animal experimentation and permitted by the responsible cantonal authorities (license N° 75668). Female athymic nude BALB/c mice and female immunocompetent FVB mice were obtained from Charles River Laboratories (Sulzfeld, Germany), at the age of 5–6 weeks. After an acclimatization period of at least 7 days, the mice were included in the studies.

### Pre-therapeutic investigation of the radioligands’ tolerability in nude mice

Eight groups of non-tumor bearing BALB/c nude mice (n = 4) with body masses in the range of 17.1–19.1 g were intravenously injected with only vehicle or the respective PSMA radioligand (10 MBq, 1 nmol per mouse) and monitored with regard to the body mass and signs of discomfort or pain (Supplementary Material). At Day 10 and Day 28, blood was sampled before euthanasia of the mice to determine albumin, blood urea nitrogen, alkaline phosphatase and total bilirubin levels using a dry chemistry analyzer (DRI-CHEM 4000i, FUJIFILM, Japan) [24]. Following euthanasia, kidneys, liver, spleen and brain were collected and weighed to determine the organ-to-brain mass ratios. The kidneys, bone marrow (sternum and femur) and spleen underwent histological assessment by a veterinarian pathologist as previously reported (Supplementary Material) [25].

### Therapy study in PC-3 PIP tumor-bearing nude mice

Six days after subcutaneous inoculation of PC-3 PIP tumor cells (4 × 10^6^ cells in 100 μL Hanks’ balanced salt solution) on the right shoulder, control mice were intravenously injected with vehicle (saline with 0.05% BSA). Three additional groups of mice were injected with [^177^Lu]Lu-Ibu-DAB-PSMA (2 MBq, 5 MBq or 10 MBq, 1 nmol per mouse), and another two groups received [^177^Lu]Lu-PSMA-617 (10 MBq, 1 nmol per mouse) or [^177^Lu]Lu-PSMA-ALB-56 (10 MBq, 1 nmol per mouse), respectively.

Data of mice injected with 2 MBq or 5 MBq (1 nmol per mouse) of [^177^Lu]Lu-PSMA-617 or [^177^Lu]Lu-PSMA-ALB-56, respectively, were obtained from previous studies performed in our group under exactly the same experimental conditions (Table 1) [16].

**Table 1:** Design of the therapy study including information about the number (n) of mice per group, the injected activity as well as the average tumor volume and average body mass of mice of each group, measured on Day 0

		Injected activity (MBq)	Tumor volume^c^ (mm^3^)	Body mass^c^ (g)
			(average ± SD)	(average ± SD)
Treatment	n		Day 0	Day 0
Saline^a^	21	-	77 ± 30	16.5 ± 1.3
[^177^Lu]Lu-Ibu-DAB-PSMA	6	2 MBq	58 ± 26	17.2 ± 1.4
[^177^Lu]Lu-Ibu-DAB-PSMA	12	5 MBq	72 ± 21	17.5 ± 1.5
[^177^Lu]Lu-Ibu-DAB-PSMA	6	10 MBq	46 ± 11	17.6 ± 1.2
[^177^Lu]Lu-PSMA-617^b^	6	2 MBq	103 ± 26	16.3 ± 1.3
[^177^Lu]Lu-PSMA-617^b^	6	5 MBq	103 ± 27	16.6 ± 0.9
[^177^Lu]Lu-PSMA-617	6	10 MBq	64 ± 15	15.6 ± 0.8
[^177^Lu]Lu-PSMA-ALB-56^b^	6	2 MBq	81 ± 28	15.3 ± 1.4
[^177^Lu]Lu-PSMA-ALB-56^b^	6	5 MBq	92 ± 37	14.9 ± 1.4
[^177^Lu]Lu-PSMA-ALB-56	6	10 MBq	63 ± 28	16.1 ± 1.6

^a^Control group (n = 15) combined with values of control mice (n = 6) from a study previously published by Umbricht et al. Mol Pharm 2018; 15:2297 − 2306. Copyright 2022 American Chemical Society [16]. ^b^Data of mice previously published in the same article of Umbricht et al. [16]. ^c^No significant differences determined between the values measured for each group (*p* > 0.05), with the exception of mice treated with 2 MBq or 5 MBq [^177^Lu]Lu-PSMA-617 compared to those treated with 10 MBq [^177^Lu]Lu-Ibu-DAB-PSMA regarding the tumor volume (*p* < 0.05)

The tumor volume (TV = 0.5 × (LW^2^) with L corresponding to the longest axis and W to the perpendicular axis) and body masses were determined every second day over 12 weeks. The relative tumor volume (RTV) was calculated as RTV = TV_x_/TV_0_, with TV_x_ corresponding to the tumor volume [mm^3^] on a given Day x, and TV_0_ corresponding to the volume [mm^3^] on Day 0. The changes in body masses over time were recorded as absolute values and relative to the body mass at therapy start, respectively (Supplementary Material). Endpoint criteria were defined as (i) > 15% body mass loss, (ii) a TV of > 800 mm^3^, (iii) a combination of body mass loss of > 10% and a TV of > 700 mm^3^ or (iv) signs of unease and pain or a combination thereof as previously reported [16]. The mice were euthanized when a predefined endpoint criterion was reached or when the study was terminated at Day 84.

The efficacy of the treatment was expressed by assessment of the tumor growth delay (TGD_x_), defined as the time required for the tumor volume to increase x-fold over the initial volume at Day 0. The tumor growth delay indices (TGDI) were calculated for a twofold (x = 2, TGD_2_) and fivefold (x = 5, TGD_5_) increase in the initial tumor volume according to the formula [TGDI_x_ = TGD_x_(T)/TGD_x_(C)] whereof TGD_x_(T) was the tumor growth delay of treated mice and TGD_x_(C) the average tumor growth delay of control mice. The survival of mice was assessed with Kaplan–Meier curves to determine the median survival of mice of each group.

### Assessment of potential impairment of blood cells in immunocompetent mice

Three groups of immunocompetent FVB mice (n = 4) were intravenously injected with [^177^Lu]Lu-Ibu-DAB-PSMA, [^177^Lu]Lu-PSMA-617 or [^177^Lu]Lu-PSMA-ALB-56 (30 MBq; 1 nmol per mouse), and an additional group of mice (n = 4) were included as untreated controls. The mice with initial body masses in the range of 19.4–23.1 g were monitored with regard to the body masses and signs of discomfort or pain (Supplementary Material). On Day 10 and Day 28 after injection, blood was sampled from the sublingual vein to determine the blood cell counts, the hematocrit and the hemoglobin concentration using a hematology analyzer (VetScan HM5, Abaxis, United States). Blood smears were prepared and stained using the Pappenheim method [26] for the analysis of morphological changes of the blood cells and counting the subgroups of leukocytes (Supplementary Material).

### Analysis and statistical methods

The data of the in vivo studies were analyzed using GraphPad Prism software (version 8) with a *p* value < 0.05 considered as the criterion for statistical significance. The values of the blood plasma parameters, body masses, organ masses and organ-to-brain mass ratios as well as hemograms and blood cell counts were assessed using a one-way ANOVA test with a Dunnett’s multiple comparison post-test. The therapy study was assessed with regard to the tumor growth and body masses using a one-way ANOVA with a Tukey’s multiple comparison post-test. The median survival of tumor-bearing mice was determined using Kaplan–Meier curves, and the statistical significance of the therapy response was analyzed using a log-rank test (Mantel Cox).

## Results

### Estimated mean absorbed dose to tumors and kidneys

The mean absorbed PC-3 PIP tumor dose for [^177^Lu]Lu-Ibu-DAB-PSMA (6.6 ± 0.8 Gy/MBq) was considerably higher than for [^177^Lu]Lu-PSMA-617 (4.5 ± 0.7 Gy/MBq) but slightly lower than for [^177^Lu]Lu-PSMA-ALB-56 (8.1 ± 1.4 Gy/MBq). The mean absorbed kidney dose for [^177^Lu]Lu-Ibu-DAB-PSMA (0.52 ± 0.06 Gy/MBq) was lower than for [^177^Lu]Lu-PSMA-ALB-56 (0.64 ± 0.13 Gy/MBq), but, in both cases, it was clearly higher than for [^177^Lu]Lu-PSMA-617 (0.070 ± 0.014 Gy/MBq). This situation resulted in equal tumor-to-kidney dose ratios for [^177^Lu]Lu-Ibu-DAB-PSMA and [^177^Lu]Lu-PSMA-ALB-56 (13 ± 3 and 13 ± 5, respectively) which was, however, fivefold lower than for [^177^Lu]Lu-PSMA-617 (64 ± 22). Considering the activity levels applied in the present study, the maximum mean absorbed kidney dose of 6.4 Gy after injection of 10 MBq [^177^Lu]Lu-PSMA-ALB-56 was still far below the estimated safe absorbed kidney dose of 23 Gy as previously determined for a folate radioconjugate (Table 2) [27].

**Table 2:** Mean absorbed dose (Gy) to PC-3 PIP tumors and kidneys calculated for the applied activity levels (MBq) of each PSMA radioligand. The values are indicated as average ± SD

	Mean absorbed PC-3 PIP tumor dose^a^			Mean absorbed kidney dose^a^		
Injected activity	2 MBq	5 MBq	10 MBq	2 MBq	5 MBq	10 MBq
[^177^Lu]Lu-Ibu-DAB-PSMA	13 ± 2	33 ± 4	66 ± 8	1.0 ± 0.1	2.6 ± 0.3	5.2 ± 0.6
[^177^Lu]Lu-PSMA-617	9.0 ± 1.3	22 ± 3	45 ± 7	0.14 ± 0.03	0.35 ± 0.07	0.70 ± 0.14
[^177^Lu]Lu-PSMA-ALB-56	16 ± 3	41 ± 7	81 ± 14	1.3 ± 0.3	3.2 ± 0.7	6.4 ± 1.3

^a^The calculations were based on non-decay-corrected biodistribution data for [^177^Lu]Lu-Ibu-DAB-PSMA (previously published by Deberle & Benešová et al. Theranostics 2020; 10:1678 − 1693 [19]), for [^177^Lu]Lu-PSMA-617 (previously published by Benešová et al. Mol Pharm 2018;15:934 − 946. Copyright 2022 American Chemical Society [11]) and for [^177^Lu]Lu-PSMA-ALB-56 (previously published by Umbricht et al. Mol Pharm 2018; 15:2297 − 2306. Copyright 2022 American Chemical Society [16]) using the same tumor mouse model

### Pre-therapeutic investigation of the radioligands’ tolerability in nude mice

In a first step, the tolerability of [^177^Lu]Lu-Ibu-DAB-PSMA was investigated for comparison with that of [^177^Lu]Lu-PSMA-617 and [^177^Lu]Lu-PSMA-ALB-56 using the same mouse strain as was subsequently used for the therapy of PC-3 PIP tumor xenografts.

**Fig. 2:**
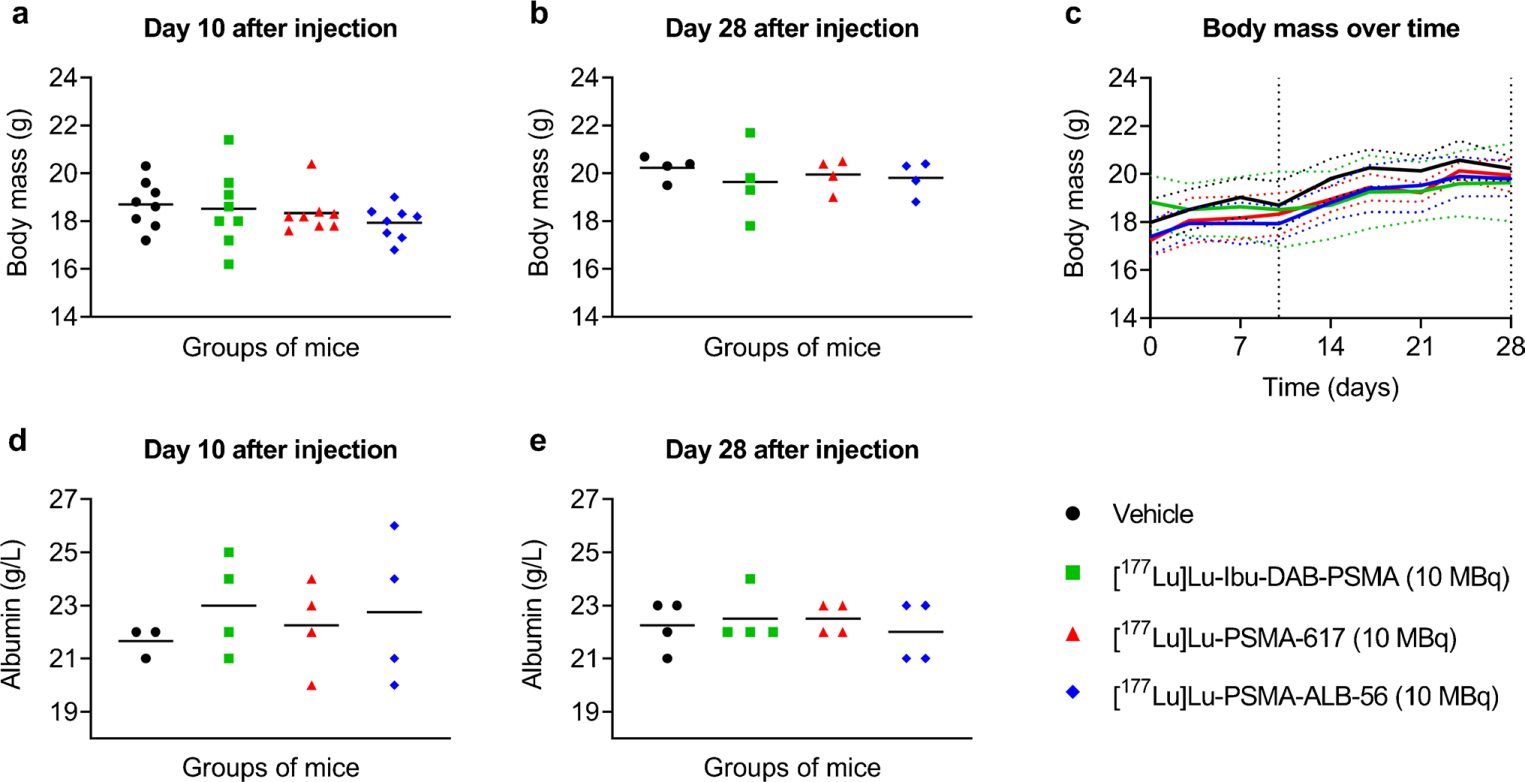
a-e Assessment of the general health status of BALB/c nude mice injected with vehicle or 10 MBq [^177^Lu]Lu-Ibu-DAB-PSMA, [^177^Lu]Lu-PSMA-617 or [^177^Lu]Lu-PSMA-ALB-56. **a/b** Body mass of mice **(a)** on Day 10 (n = 8) or **(b)** on Day 28 (n = 4). **c** Body mass monitored during the study (the vertical dashed lines correspond to Day 10 and Day 28). **d/e** Albumin concentration in blood plasma measured in mice **(d)** on Day 10 or **(e)** on Day 28

All groups of non-tumor-bearing BALB/c nude mice injected with either vehicle, or PSMA radioligand (10 MBq, 1 nmol per mouse) were healthy and did not show any signs of pain or unease during the entire period of investigation. At the beginning of the study, the body mass of mice that received [^177^Lu]Lu-PSMA-617 and [^177^Lu]Lu-PSMA-ALB-56 was somewhat lower than for those injected with [^177^Lu]Lu-Ibu-DAB-PSMA; however, all mice had similar body masses on Day 10 and 28 after injection of the radioligands or vehicle only (*p* > 0.05) (Fig. 2a-c; Supplementary Material, Table S1). The blood plasma concentration of serum albumin, an indicator of the general health status of the mice, was equal in all mice at both investigated timepoints (*p* > 0.05) (Fig. 2d/e; Supplementary Material, Table S2).

**Fig. 3:**
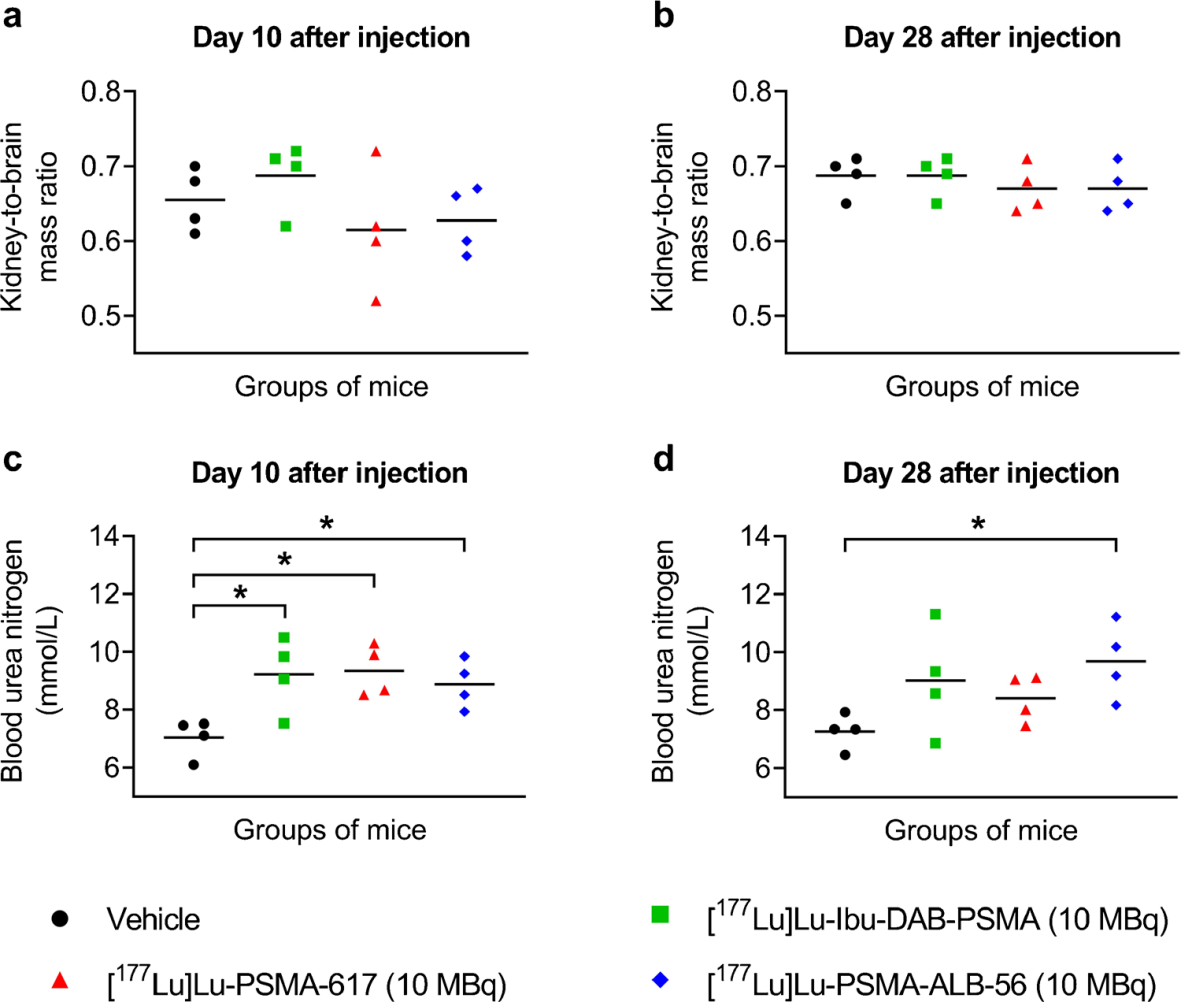
a-d Assessment of potential early undesired effects to the kidneys in BALB/c nude mice injected with vehicle or 10 MBq [^177^Lu]Lu-Ibu-DAB-PSMA, [^177^Lu]Lu-PSMA-617 or [^177^Lu]Lu-PSMA-ALB-56. **a/b** Kidney-to-brain mass ratios **(a)** on Day 10 or **(b)** on Day 28. **c/d** Blood urea nitrogen concentrations measured in mice **(c)** on Day 10 or **(d)** on Day 28. *significantly different from control group (*p* < 0.05)

The brain mass is generally constant in adult mice irrespective of the health status; therefore, the organ mass-to-brain mass ratios can be used as an indicator for the health condition of the mice. The kidney masses relative to the brain masses, indicated as kidney-to-brain mass ratios, revealed values in the same range for mice injected with any of the PSMA radioligands or vehicle only (Fig. 3a/b; Supplementary Material, Table S1). The histological assessment of the renal tissue did not reveal any abnormalities in any of the mice, irrespective of the group to which they belonged (Supplementary Material, Fig. S2). Blood urea nitrogen levels, a measure for renal function, were significantly higher in mice injected with the radioligands than in control mice on Day 10 (*p* < 0.05) (Fig. 3c; Supplementary Material, Table S2). On Day 28, the difference in blood urea nitrogen concentrations was still significant (*p* < 0.05) between mice injected with [^177^Lu]Lu-PSMA-ALB-56 and untreated control mice, but for [^177^Lu]Lu-Ibu-DAB-PSMA or [^177^Lu]Lu-PSMA-617 only a trend of increased blood urea nitrogen values was observed (*p* > 0.05) (Fig. 3d). According to values for normal blood urea nitrogen levels in BALB/c nude mice listed by Charles River Laboratories, all measured concentrations were still in the physiological range; hence, it is unlikely that the obtained results indicates a sign of impaired kidney function.

Liver-to-brain and spleen-to-brain mass ratios were comparable among mice treated with either [^177^Lu]Lu-Ibu-DAB-PSMA or [^177^Lu]Lu-PSMA-ALB-56 and those that received [^177^Lu]Lu-PSMA-617 or only vehicle (Supplementary Material, Table S1). The only exception was an increased liver-to-brain mass ratio observed on Day 28 for mice that received [^177^Lu]Lu-PSMA-617 (*p* < 0.05). Plasma levels of alkaline phosphatase and total bilirubin were all in the same range for mice injected with a radioligand and control mice. The histopathological analysis of the spleen and the bone marrow did not show abnormalities in any of the mice included in this study (Supplementary Material, Figs. S3/S4).

### Therapeutic efficacy of [^177^Lu]Lu-Ibu-DAB-PSMA in comparison with other radioligands

**Fig. 4:**
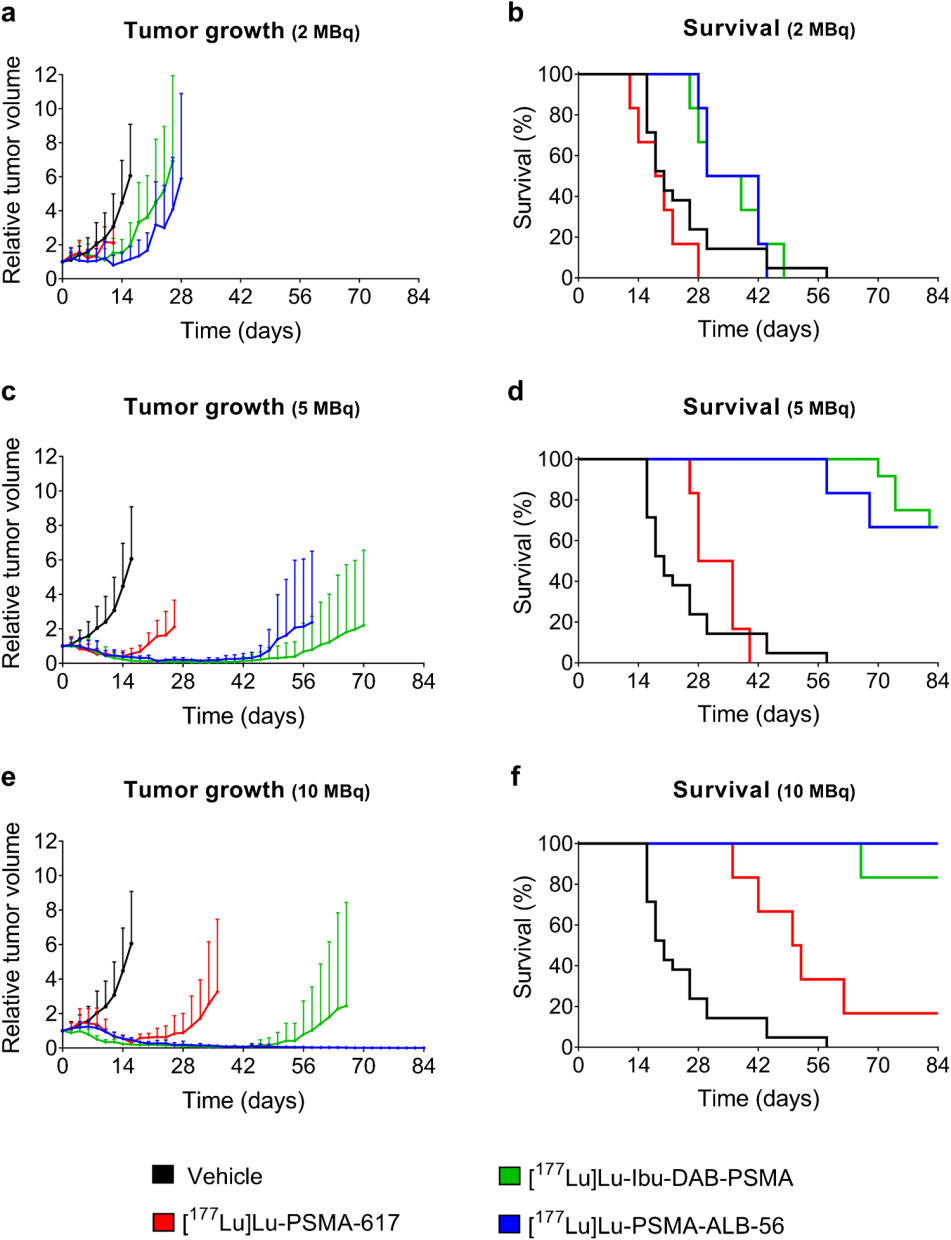
a-f Relative tumor growth curves (shown until the first mouse reached an endpoint) and survival curves of control mice and mice treated with different activities of PSMA radioligands (average ± SD, n ≥ 6). **a/b** Application of 2 MBq; **c/d** Application of 5 MBq; **e/f** Application of 10 MBq radioligand. Tumor growth curves and survival curves of mice injected with vehicle are based on data obtained in this study combined with values from a previous study published by Umbricht et al. Mol Pharm 2018; 15:2297 − 2306. Copyright 2022 American Chemical Society [16]. Tumor growth curves of mice injected with [^177^Lu]Lu-PSMA-617 (2 MBq and 5 MBq) and [^177^Lu]Lu-PSMA-ALB-56 (2 MBq and 5 MBq) are based on previously obtained data, published in the same article by Umbricht et al. [16]

Tumors of untreated control mice increased in size constantly over time so that the first mouse of this group reached the endpoint at Day 16 (Fig. 4; Table 3). [^177^Lu]Lu-Ibu-DAB-PSMA applied at 2 MBq delayed the tumor growth moderately, demonstrated by a TGDI_2_ and TGDI_5_ of 1.3 ± 0.7 and 1.5 ± 0.4, respectively. [^177^Lu]Lu-PSMA-ALB-56 applied at the same activity showed an even slightly increased tumor growth delay (TGDI_2_ of 1.9 ± 0.6 and TGDI_5_ of 1.7 ± 0.5, respectively). The application of the same activity of [^177^Lu]Lu-PSMA-617 did, however, not show any significant tumor growth inhibition as compared to control values, demonstrated by TGDIs in the range of 1.0–1.1 (Fig. 4a; Table 3). As a result, the survival of mice treated with 2 MBq [^177^Lu]Lu-PSMA-617 (median survival of 19 days) was not increased as compared to the survival of control mice (median survival of 20 days) (*p* > 0.05). In contrast, mice treated with 2 MBq [^177^Lu]Lu-Ibu-DAB-PSMA or [^177^Lu]Lu-PSMA-ALB-56 survived significantly longer (*p* < 0.05) demonstrated by much longer median survival times of 34 days and 36 days, respectively. (Fig. 4b).

**Table 3:** Parameters indicative for the efficacy of the treatment, including the day when the first and last mouse of each group were euthanized, median survival and tumor growth delay indices

Treatment	Injected activity	First mouse euthanized	Last mouse euthanized	Median Survival	TGDI_2_	TGDI_5_
	(MBq)	(Day)	(Day)	(Day)		
Saline	-	16	58	20	1.0 ± 0.8	1.0 ± 0.5
[^177^Lu]Lu-Ibu-DAB-PSMA	2	26	48	34	1.3 ± 0.7	1.5 ± 0.4
[^177^Lu]Lu-Ibu-DAB-PSMA	5	70	84^b^	> > 84^c^	> > 6.0^e^	> > 4.6^e^
[^177^Lu]Lu-Ibu-DAB-PSMA	10	66	84^b^	> > 84^c^	> > 6.1^e^	> > 4.6^e^
[^177^Lu]Lu-PSMA-617^a^	2	12	28	19	1.0 ± 0.3^d^	1.1 ± 0.1^d^
[^177^Lu]Lu-PSMA-617^a^	5	26	40	32	2.1 ± 0.4^d^	1.9 ± 0.3^d^
[^177^Lu]Lu-PSMA-617	10	36	84^b^	51	3.1 ± 1.0	2.7 ± 0.7
[^177^Lu]Lu-PSMA-ALB-56^a^	2	28	44	36	1.9 ± 0.6^d^	1.7 ± 0.5^d^
[^177^Lu]Lu-PSMA-ALB-56^a^	5	58	84^b^	> > 84^c^	> > 5.7^d, e^	> > 4.3^d, e^
[^177^Lu]Lu-PSMA-ALB-56	10	84^b^	84^b^	> > 84^c^	> > 6.5^e^	> > 4.9^e^

^a^Data of mice previously published by Umbricht et al. Mol Pharm 2018; 15:2297 − 2306. Copyright 2022 American Chemical Society [16]. ^b^All mice were euthanized at the end of the study at Day 84 even if not all mice had reached an endpoint. ^c^The exact median survival could not be defined, since more than half of the mice survived until endpoint. ^d^Values recalculated based on data obtained from the control group that comprised mice from this study (n = 15) and mice (n = 6) from the study, previously published in the same article published by Umbricht et al.[16]. ^e^The exact TGDI values could not be determined because the respective groups did not even reach a RTV of 2

The difference in efficacy between the albumin-binding radioligands and [^177^Lu]Lu-PSMA-617 was even more pronounced after administration of higher activities (Fig. 4c). Most mice treated with 5 MBq [^177^Lu]Lu-Ibu-DAB-PSMA or 5 MBq [^177^Lu]Lu-PSMA-ALB-56 did not even reach a RTV of 2. In these mice, almost all tumors had disappeared after about 7 weeks. In 4/12 mice treated with [^177^Lu]Lu-Ibu-DAB-PSMA and 2/6 mice treated with [^177^Lu]Lu-PSMA-ALB-56, the tumors started, however, to regrow after a certain time. While the application of 5 MBq [^177^Lu]Lu-PSMA-617 resulted in a median survival time of 32 days, median survival times could not be determined for the other groups as 8/12 mice (67%) treated with 5 MBq [^177^Lu]Lu-Ibu-DAB-PSMA and 4/6 mice (67%) treated with 5 MBq [^177^Lu]Lu-PSMA-ALB-56 survived until the end of the study (Fig. 4d).

The tumors were effectively eradicated in 5/6 mice (83%) treated with 10 MBq [^177^Lu]Lu-Ibu-DAB-PSMA so that these mice survived until study end without regrowth of the tumor. Just in one case, the tumor started to regrow about 7 weeks after the treatment, about one month later than for mice treated with 10 MBq [^177^Lu]Lu-PSMA-617 (Fig. 4e/f). Mice treated with 10 MBq [^177^Lu]Lu-PSMA-617 had a median survival time of 51 days with only 1/6 mouse alive (17%) at study end (Fig. 4f). Application of 10 MBq [^177^Lu]Lu-PSMA-ALB-56 resulted in eradication of tumors in 6/6 mice (100%) so that these mice were all alive at study end on Day 84 (Fig. 4e/f).

The body mass of mice treated with 10 MBq [^177^Lu]Lu-PSMA-617 and of mice treated with 5 MBq or 10 MBq [^177^Lu]Lu-Ibu-DAB-PSMA or [^177^Lu]Lu-PSMA-ALB-56 increased over the course of the therapy study. The fast tumor growth in mice of all other groups affected the overall health condition of the mice indicated by only marginal gain in body mass over time or—in some cases—even loss of body mass. At the time of euthanasia, the average body mass of these groups was significantly lower (*p* < 0.05) than in the other groups (Supplementary Material, Fig. S5). In some cases, the loss of body mass was even the relevant criterion that mice reached the endpoint.

### Assessment of potential impairment of blood cells in immunocompetent mice

**Fig. 5:**
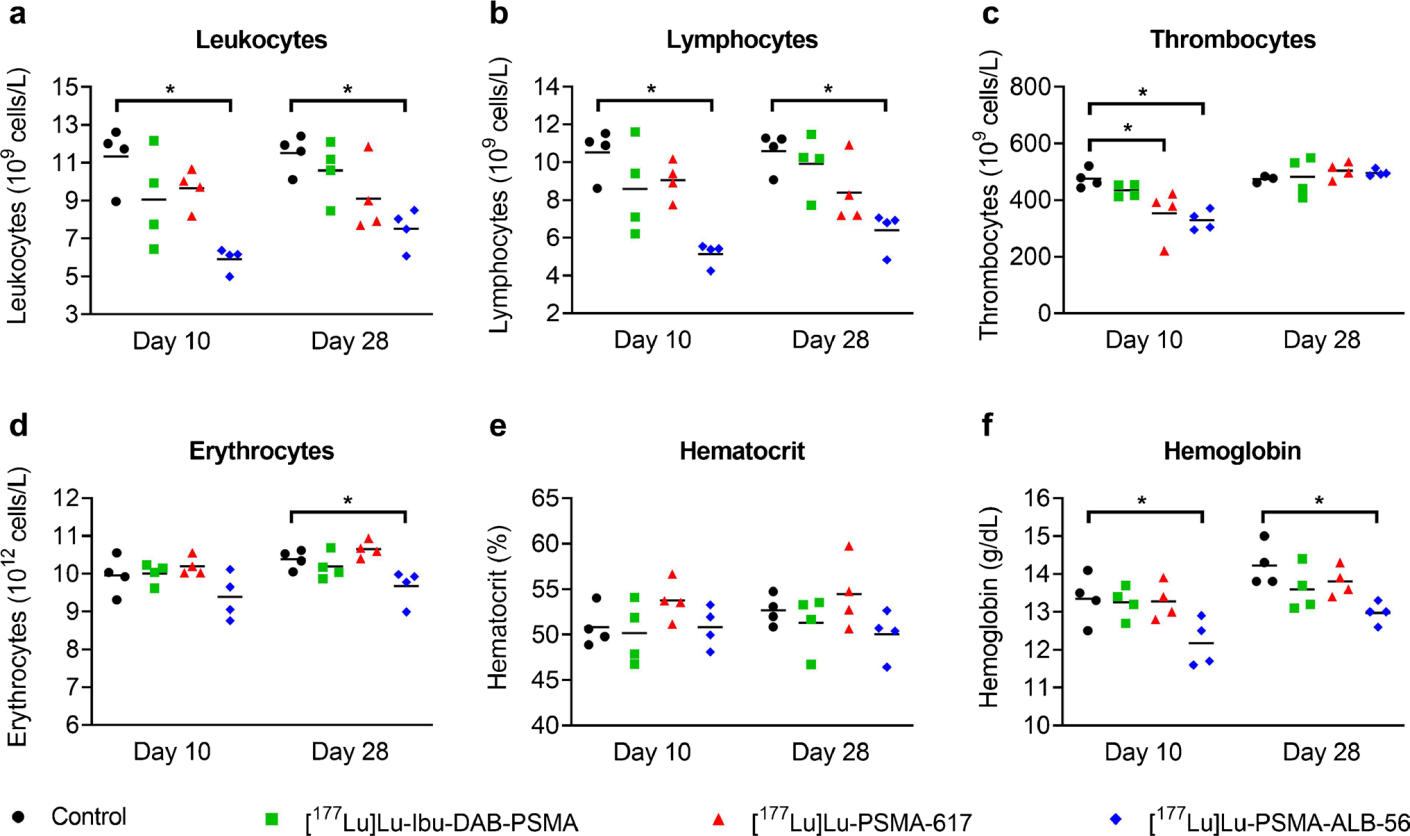
a-f Analysis of the blood cell counts, hematocrit and hemoglobin concentrations of immunocompetent FVB mice (n = 4) on Day 10 and Day 28 after application of 30 MBq [^177^Lu]Lu-IbuDAB-PSMA, [^177^Lu]Lu-PSMA-617 or [^177^Lu]Lu-PSMA-ALB-56 in comparison with control mice. **a** Leukocyte counts; **b** Lymphocyte counts; **c** Thrombocyte counts; **d** Erythrocyte counts; **e** Hematocrit; **f** Hemoglobin concentrations. *significantly different from control group (*p* < 0.05)

At both investigated timepoints (Day 10 and Day 28), the mice injected with 30 MBq [^177^Lu]Lu-Ibu-DAB-PSMA showed no significant hematological changes compared to mice of the control group (*p* > 0.05) (Fig. 5). On Day 10 after injection of [^177^Lu]Lu-PSMA-ALB-56, the leukocyte and lymphocyte counts dropped to (5.9 ± 0.6) × 10^9^/L and (5.1 ± 0.6) × 10^9^/L, respectively, which was significantly lower (*p* < 0.05) than the respective blood cell counts of control mice ((11 ± 2) × 10^9^/L and (11 ± 1) × 10^9^/L) (Fig. 5a/b). On Day 28, the difference in leukocyte and lymphocyte counts between mice treated with [^177^Lu]Lu-PSMA-ALB-56 and untreated control mice was still significant (*p* < 0.05). Although the leukocyte counts of mice that received [^177^Lu]Lu-Ibu-DAB-PSMA or [^177^Lu]-PSMA-617 were slightly lower than the values of control mice, the differences were not significant (*p* > 0.05) at either of the investigated timepoints. The blood smear-based analysis of the leukocyte subgroups revealed a decreased percentage of lymphocytes and an increased percentage of neutrophils for mice treated with [^177^Lu]Lu-PSMA-ALB-56 as compared to controls on Day 28, which was in contrast to the other groups that showed values in the same range as controls (Supplementary Material, Table S3). On Day 10, thrombocyte counts were comparable for mice that received [^177^Lu]Lu-Ibu-DAB-PSMA and control mice, but significantly lower (*p* < 0.05) for [^177^Lu]Lu-PSMA-617 and [^177^Lu]Lu-PSMA-ALB-56; however, on Day 28, thrombocyte counts were in the same range for all groups (Fig. 5c). On Day 10, no significant changes in erythrocyte counts or hematocrit were observed in treated mice as compared to untreated controls (Fig. 5d/e), but for mice injected with [^177^Lu]Lu-PSMA-ALB-56, the hemoglobin concentration was lower at this timepoint. On Day 28, mice that received [^177^Lu]Lu-Ibu-DAB-PSMA or [^177^Lu]Lu-PSMA-617 showed erythrocyte counts and hemoglobin concentrations in the same range as control mice ((10.2–10.7) × 10^12^/L and 13.6–14.2 g/dL, respectively); however, these values were significantly lower in mice injected with [^177^Lu]Lu-PSMA-ALB-56 ((9.7 ± 0.5) × 10^12^/L and 13.0 ± 0.3 g/dL, respectively) (Fig. 5d/f). The analysis of blood smears revealed no morphological changes of blood cells from any of the treated mice nor from control mice. During the course of this study, none of the treated groups showed any significant changes in average body mass compared to mice of the control group (Supplementary Material, Fig. S6).

## Discussion

As demonstrated in a pre-therapeutic tolerability study, [^177^Lu]Lu-Ibu-DAB-PSMA was well tolerated in BALB/c nude mice at an activity of 10 MBq per mouse and did not reveal any undesired early effects on normal tissue over the investigated time period of 28 days. The average body mass of mice of all groups was similar on Day 10 and Day 28 after injection of the respective radioligand (*p* > 0.05), irrespective of variations of the average body mass between the groups on Day 0. The blood plasma parameters were in the physiological range for all mice, and the histopathological examination of the kidneys, spleen and bone marrow did not reveal any differences among mice injected with a radioligand or with only vehicle, indicating that all radioligands were well tolerated. It was, thus, concluded that 10 MBq [^177^Lu]Lu-Ibu-DAB-PSMA or 10 MBq [^177^Lu]Lu-PSMA-ALB-56 can be safely applied for the treatment of tumor-bearing mice. This was still below the maximum activity applied by other groups who tested ^177^Lu-based RLT in mice using albumin-binding PSMA ligands or PSMA-617 in immunodeficient [14, 28, 29] and immunocompetent mice [30].

The therapeutic efficacy of [^177^Lu]Lu-Ibu-DAB-PSMA in tumor-bearing nude mice was compared to the effect of [^177^Lu]Lu-PSMA-617, applied at the same activity levels and also set into relation with the therapy data obtained for the previously developed [^177^Lu]Lu-PSMA-ALB-56 [16]. In line with the increased absorbed tumor dose of the albumin-binding radioligands, the tumor growth inhibition was much more pronounced after application of [^177^Lu]Lu-Ibu-DAB-PSMA and [^177^Lu]Lu-PSMA-ALB-56 than after injection of [^177^Lu]Lu-PSMA-617. At the lowest activity (2 MBq/mouse), the difference in efficacy between [^177^Lu]Lu-Ibu-DAB-PSMA and [^177^Lu]Lu-PSMA-ALB-56 was not as obvious as it could have been expected based on the higher calculated absorbed tumor dose for [^177^Lu]Lu-PSMA-ALB-56. [^177^Lu]Lu-Ibu-DAB-PSMA injected at 5 MBq per mouse was effective in delaying the tumor growth in mice. The benefit of using 10 MBq/mouse was only visible from the increased number of mice (83% vs. 67%) that survived until the end of the study. The improved therapeutic efficacy of [^177^Lu]Lu-PSMA-ALB-56 as compared to [^177^Lu]Lu-Ibu-DAB-PSMA was observed when comparing the data of mice injected with 10 MBq radioligand, which showed complete eradication of tumors in mice treated with [^177^Lu]Lu-PSMA-ALB-56 but not in mice that received [^177^Lu]Lu-Ibu-DAB-PSMA.

Radiopharmaceuticals with commonly high kidney uptake, including folate radioconjugates or glucagon-like peptide-1-based radiopeptides, showed reduced renal retention after modification with an albumin binder [31–33]. This was, however, not the case for [^177^Lu]Lu-Ibu-DAB-PSMA and [^177^Lu]Lu-PSMA-ALB-56 or any other albumin-binding PSMA radioligand [10, 12, 14, 28], which all showed higher kidney retention than [^177^Lu]Lu-PSMA-617. The reason for this observation remains unexplored, but apparently, certain structural elements of these radioligands favor retention in the kidneys. In the case of [^177^Lu]Lu-PSMA-ALB-56, the calculated absorbed kidney dose of 0.64 ± 0.13 Gy/MBq in mice translated to a kidney dose of 2.54 ± 0.94 Gy/GBq in patients [18]. Since the absorbed kidney dose in mice was 0.52 ± 0.06 Gy/MBq for [^177^Lu]Lu-Ibu-DAB-PSMA, it can be speculated that the kidney dose in patients would be in the range of 2 Gy/GBq. This is still significantly higher than for [^177^Lu]Lu-PSMA-617 (0.39 ± 0.15 Gy/GBq) [18], but would possibly enable a safe application of several therapy cycles, in particular because the administered activity per cycle would most probably be lower than commonly used for [^177^Lu]Lu-PSMA-617.

Importantly, the AUC_0→192 h_ values indicated a 2.6-fold reduced blood retention for [^177^Lu]Lu-Ibu-DAB-PSMA as compared to that of [^177^Lu]Lu-PSMA-ALB-56 (Supplementary Material, Fig. S7, Table S4) [19]. It is, thus, expected that the mean absorbed bone marrow dose after application of [^177^Lu]Lu-Ibu-DAB-PSMA would be significantly lower than in the case of [^177^Lu]Lu-PSMA-ALB-56, for which the bone marrow was considered the dose-limiting organ [18]. In a study with immunocompetent mice, it was confirmed that hematological parameters including blood cell counts of mice treated with 30 MBq [^177^Lu]Lu-Ibu-DAB-PSMA were similar to those treated with 30 MBq [^177^Lu]Lu-PSMA-617 or control mice. These values were commonly higher than those reported in the literature for FVB mice [34], which can be ascribed to the blood sampling from the sublingual vein applied in our study. Importantly, the application of [^177^Lu]Lu-PSMA-ALB-56 at the same activity resulted in a transient decrease in erythrocyte counts, hemoglobin concentration and lymphocyte counts.

Other research groups did not observe severe hematotoxicity in immunocompetent mice after injection of up to sixfold higher activities of [^177^Lu]Lu-PSMA-617 [30, 35]. It has to be critically acknowledged that the application of such high activities would most probably not be possible without causing undesired hematological effects when using albumin-binding PSMA radioligands.

In patients, the salivary glands are a tissue at risk of radiotoxicity after RLT, in particular when using actinium-225 for targeted α-therapy [36–38]. Interestingly, clinical application of [^177^Lu]Lu-PSMA-ALB-56 did not show increased salivary gland uptake as compared to [^177^Lu]Lu-PSMA-617 [18]. This may indicate an advantage of using albumin-binding radioligands in order to achieve a sufficiently high tumor-to-salivary gland dose ratio. The salivary gland uptake of radioligands in mice is, however, not predictive for the situation in human patients, and, hence, a potential advantage of [^177^Lu]Lu-Ibu-DAB-PSMA over [^177^Lu]Lu-PSMA-617 in this regard remains to be demonstrated in a clinical setting.

## Conclusion

The results of this study confirmed the anticipated therapeutic superiority of [^177^Lu]Lu-Ibu-DAB-PSMA over [^177^Lu]Lu-PSMA-617 in tumor-bearing mice. At the same time, [^177^Lu]Lu-Ibu-DAB-PSMA did not affect blood cell counts at an activity level that resulted in changes in hematological parameters when using [^177^Lu]Lu-PSMA-ALB-56. Translation of [^177^Lu]Lu-Ibu-DAB-PSMA to a clinical setting is, thus, warranted to shed light on its utility to treat prostate cancer patients and its future applicability in nuclear oncology.

## Supplementary Information

Below is the link to the electronic supplementary material.

Supplementary file1 (DOCX 15.4 mb)

## Data Availability

The raw data of the results presented in this study are available on request from the corresponding author.

## Abbreviations

ANOVA: Analysis of variance
AUC: Area under the curve
BSA: Bovine serum albumin
DAB: Diaminobutyric acid
Ibu: ibuprofen
mCRPC: Metastatic castration-resistant prostate cancer
PSMA: Prostate-specific membrane antigen
RLT: Radioligand therapy
RTV: Relative tumor volume
SD: Standard deviation
TGD: Tumor growth delay
TGDI: Tumor growth delay index
TIACCs: Time integrated activity concentration coefficients
TV: Tumor volume
