# Supplementary material for: Preclinical investigations using [177Lu]Lu-Ibu-DAB-PSMA toward its clinical translation for radioligand therapy of prostate cancer
Source: Eur J Nucl Med Mol Imaging. 2022 May 30;49(11):3639–50. doi: 10.1007/s00259-022-05837-2 (PMC9399046; doi:10.1007/s00259-022-05837-2)
Supplement: Supplementary file 1 — Supplementary file1 (DOCX 15.4 mb) [file 259_2022_5837_MOESM1_ESM.docx]

SUPPLEMENTARY MATERIAL

**Preclinical investigations using [^177^Lu]Lu-Ibu-DAB-PSMA toward its clinical translation for radioligand therapy of prostate cancer**

Viviane J. Tschan^1^, Francesca Borgna^1^, Sarah D. Busslinger^1^, Martina Stirn^2^, Josep M. Monné Rodriguez^3^, Peter Bernhardt^4^, Roger Schibli^1, 5^, Cristina Müller^1, 5*^

1. Center for Radiopharmaceutical Sciences ETH-PSI-USZ, Paul Scherrer Institute, 5232 Villigen-PSI, Switzerland

2. Clinical Laboratory, Department of Clinical Diagnostics and Services, Vetsuisse Faculty, University of Zurich, 8057 Zurich, Switzerland

3. Laboratory for Animal Model Pathology (LAMP), Institute of Veterinary Pathology, Vetsuisse Faculty, University of Zurich, 8057 Zurich, Switzerland

4. Department of Radiation Physics, Institution of Clinical Science, Sahlgrenska Academy, University of Gothenburg, 41345 Gothenburg, Sweden

5. Department of Chemistry and Applied Biosciences, ETH Zurich, 8093 Zurich, Switzerland

***Correspondence to**:

PD Dr. Cristina Müller

Center for Radiopharmaceutical Sciences ETH-PSI-USZ

Paul Scherrer Institute

5232 Villigen-PSI

Switzerland

e-mail: cristina.mueller@psi.ch

phone: +41-56-310 44 54; fax: +41-56-310 28 49

**1. Dosimetry calculations**

**Purpose:** Dosimetry calculations were performed in order to estimate the difference of the absorbed tumor and kidney doses for [^177^Lu]Lu-Ibu-DAB-PSMA, [^177^Lu]Lu-PSMA-617 and [^177^Lu]Lu-PSMA-ALB-56, respectively.

**Methods:** Dosimetry calculations were performed to estimate the mean specific absorbed dose of the tumor and the kidneys and compare the values for the three investigated radioligands. Bi-exponential functions were used to describe the tissue biokinetics of [^177^Lu]Lu-Ibu-DAB-PSMA, [^177^Lu]Lu-PSMA-617 and [^177^Lu]Lu-PSMA-ALB-56. Uncertainties in the time-integrated activity concentration coefficients (TIACCs) were generated by using the mean ± the standard deviation (SD) for the measured tissue activity concentrations. For each biokinetic data set, a bi-exponential curve fit was performed using the software MATLAB (MathWorks, Torrance, California, USA). The TIACCs were obtained by integrating the generated bi-exponential functions to infinity. The specific mean absorbed dose *D* for the PC-3 PIP tumor and kidneys was calculated by:

$D=TIACC\cdot\left( \sum_{i} {E_{i}\gamma_{i}\emptyset}_{i} \right)$ (1)

where *E_i_* is the energy emitted of the *i*^th^ radiation with a frequency per decay of *γ*_i_; *∅_i_* the absorbed energy fraction within an organ. The absorbed fractions were calculated by Monte Carlo simulation using PENELOPE 2014 [1]. In the simulations, spherical shapes of the organs were assumed. The decay data of lutetium-177 were obtained from ICRU 107 ([www.nucleide.org](http://www.nucleide.org)).

**Results:** The results are reported in the main article.

**2. Radiolabeling and quality control**

**Purpose:** Ibu-DAB-PSMA [2], PSMA-617 [3] and PSMA-ALB-56 [4] were labeled with lutetium-177 for the experiments reported in this article.

**Methods:** Ibu-DAB-PSMA, PSMA-617 and PSMA-ALB-56 were labeled with no-carrier-added lutetium-177 ([^177^Lu]LuCl_3_/HCl (0.04 M); ITM Medical Isotopes GmbH, Germany) in a mixture of sodium acetate (0.5 M, pH ~8) and HCl (0.05 M) at pH 4.5 to obtain a molar activity of up to 50 MBq/nmol. The reaction mixtures were incubated for 10 min at 95 °C. Quality control of the radiolabeled PSMA ligands was performed using a Merck Hitachi LaChrom high performance liquid chromatography (HPLC) system, equipped with a D-7000 interface, a L-7200 autosampler, a radioactivity detector (LB 506 B from Berthold, Germany) and a L-7100 pump connected to a reversed-phase C18 column (5 µm, 150×4.6 mm, Xterra^TM^, MS, Waters, USA). The mobile phase consisted of 0.1% trifluoroacetic acid in Milli-Q water and acetonitrile using a linear gradient of 95–20% water phase over 15 min at a flow rate of 1 mL/min. Quality control was performed by injection of a sample of the labeling mixture diluted in water containing pentasodium diethylenetriaminepentaacetate (Na_5_-DTPA; 50 µM).

**Results:** The quality control of the radiolabeled PSMA ligands (50 MBq/nmol) showed >98% radiochemical purity (Fig. S1). In the studies reported in this article, the radioligands were applied without purification at a maximum molar activity of 30 MBq/nmol.


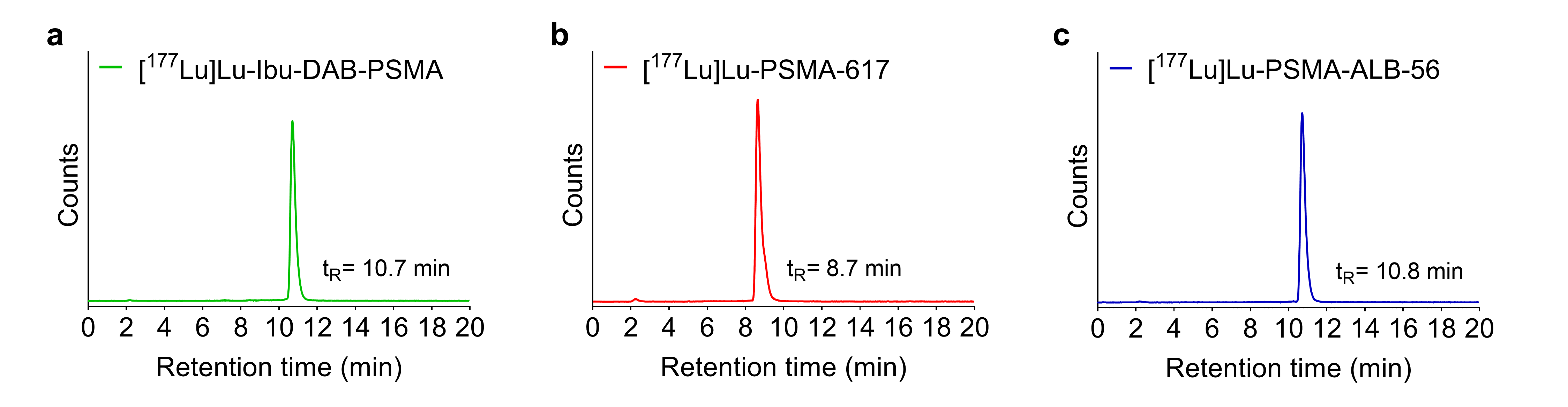


**Fig. S1** **a-c** Representative chromatograms of the radiolabeled PSMA ligands (50 MBq/nmol). The product peaks and their retention times (t_R_) are shown for **(a)** [^177^Lu]Lu-Ibu-DAB-PSMA, **(b)** [^177^Lu]Lu-PSMA-617 and **(c)** [^177^Lu]Lu-PSMA-ALB-56. The presence of uncoordinated lutetium-177 detected as [^177^Lu]Lu-DTPA would appear with a retention time of t_R_ = 2.4 ± 0.2 min.

**3. Pre-therapeutic investigation of the radioligands’ tolerability in nude mice**

**Purpose:** The tolerability of the radioligands was investigated at 10 MBq per mouse, the highest activity planned to be used in the preclinical therapy study with tumor-bearing BALB/c nude mice.

**Methods:** BALB/c nude mice were intravenously injected with only vehicle or 10 MBq of the respective PSMA radioligand. Mice were monitored by measuring the body mass twice a week over a period of 10 days and 28 days, respectively. Endpoint criteria were defined as (i) >15% loss in body mass, (ii) signs of unease and pain or a combination thereof. The mice were euthanized on Day 10 or on Day 28, respectively. In order to evaluate the tolerability of the radioligands, blood plasma parameters were determined and the body mass and organ masses as well as the corresponding organ-to-brain mass ratios were calculated next to the histological assessment of selected organs and tissues.

*Histopathological assessment of organs:* The kidneys, bone marrow (sternum and femur), and spleen were collected for histological assessment as previously reported [5]. The tissue was fixed in 4% neutral-buffered formalin and embedded in paraffin wax. Before paraffin embedding, sternum and femur tissues were decalcified at room temperature in an ethylenediaminetetraacetic acid (EDTA)-citrate solution for 2 days. Sections of 3–5 μm thickness were prepared and stained with hematoxylin eosin. Histological lesions were semi-quantitatively scored by a veterinary pathologist in a blinded manner using a severity grading scheme that ranged from 0 to 5, discriminating no lesions (score 0), minimal (score 1), mild (score 2), moderate (score 3), moderate to severe (score 4) and severe damage (score 5). Renal microscopic changes of the glomerular, tubular, and interstitial compartments were scored separately and the sum of the three values was used to obtain a cumulative score representing potential radiation nephropathy damage, as previously described [6]. Histological evaluation of the spleen was conducted to assess potential radiation injury in the lymphoid cells of the white pulp. A scoring system from 0–5 was used with a score of 0 indicating no lymphoid depletion and a score of 5 indicating severe lymphoid depletion. The extra-medullary hematopoiesis (EMH) of the red pulp was also scored from 0–5 using a score of 0 indicating no EMH to a score of 5, which indicated large numbers of EMH precursors. The hematopoietic function was also evaluated in the bone marrow by estimating the overall cellularity using a scoring from 0–5 with a score of 0 indicating no reduction in cellularity up to a score of 5 indicating severe reduction in cellularity and alteration of the proportion of the different cellular lineages (including granulocytic precursors, erythroid cells and megakaryocytes).

**Results:** The most relevant results of the pre-therapeutic tolerability study are reported in the main article. On Day 28 after application, kidney masses were lower in the group that received [^177^Lu]Lu-Ibu-DAB-PSMA, however, the similar kidney-to-brain ratios clearly demonstrated that this was a result of smaller mice in this group rather than of a change in kidney mass over time (Table S1).

**Table S1** The absolute body masses and organ masses and respective mass ratios determined on Day 10 and Day 28 after injection of 10 MBq radioligand

| **Day 10** | | | | | | | |
| --- | --- | --- | --- | --- | --- | --- | --- |
| **Group**  **n=4** | **Body mass**  **(g)** | **Kidney mass**  **(mg)** | | **Liver mass**  **(mg)** | **Spleen mass**  **(mg)** | | **Brain mass**  **(mg)** |
| Vehicle | 18.7 ± 1.1 | 276 ± 13 | | 1100 ± 132 | 105 ± 8 | | 422 ± 23 |
| [^177^Lu]Lu-Ibu-DAB-PSMA | 19.3 ± 1.5 | 271 ± 31 | | 1138 ± 80 | 106 ± 16 | | 394 ± 25 |
| [^177^Lu]Lu-PSMA-617 | 18.8 ± 1.1 | 251 ± 28 | | 1108 ± 78 | 105 ± 15 | | 410 ± 15 |
| [^177^Lu]Lu-PSMA-ALB-56 | 17.7 ± 0.7 | 247 ± 18 | | 1022 ± 74 | 87 ± 5 | | 393 ± 18 |
| **Day 28** | | | | | | | |
| **Group**  **n=4** | **Body mass**  **(g)** | **Kidney mass**  **(mg)** | | **Liver mass**  **(mg)** | **Spleen mass**  **(mg)** | | **Brain mass**  **(mg)** |
| Vehicle | 20.2 ± 0.5 | 299 ± 16 | | 1053 ± 103 | 106 ± 7 | | 434 ± 18 |
| [^177^Lu]Lu-Ibu-DAB-PSMA | 19.7 ± 1.6 | 252 ± 34^*^ | | 1046 ± 112 | 108 ± 18 | | 401 ± 8^*^ |
| [^177^Lu]Lu-PSMA-617 | 20.0 ± 0.7 | 267 ± 9 | | 1153 ± 34 | 125 ± 30 | | 398 ± 13^*^ |
| [^177^Lu]Lu-PSMA-ALB-56 | 19.8 ± 0.7 | 288 ± 22 | | 1144 ± 99 | 102 ± 18 | | 405 ± 25 |
| **Day 10** | | | | | | | |
| **Group**  **n=4** | **Kidney-to-brain mass ratios** | | **Liver-to-brain mass ratios** | | | **Spleen-to-brain mass ratios** | |
| Vehicle | 0.654 ± 0.043 | | 2.614 ± 0.371 | | | 0.250 ± 0.018 | |
| [^177^Lu]Lu-Ibu-DAB-PSMA | 0.686 ± 0.046 | | 2.887 ± 0.086 | | | 0.269 ± 0.033 | |
| [^177^Lu]Lu-PSMA-617 | 0.614 ± 0.081 | | 2.707 ± 0.240 | | | 0.257 ± 0.041 | |
| [^177^Lu]Lu-PSMA-ALB-56 | 0.629 ± 0.045 | | 2.610 ± 0.264 | | | 0.221 ± 0.013 | |
| **Day 28** | | | | | | | |
| **Group**  **n=4** | **Kidney-to-brain mass ratios** | | **Liver-to-brain mass ratios** | | | **Spleen-to-brain mass ratios** | |
| Vehicle | 0.689 ± 0.024 | | 2.422 ± 0.170 | | | 0.243 ± 0.006 | |
| [^177^Lu]Lu-Ibu-DAB-PSMA | 0.628 ± 0.075 | | 2.608 ± 0.251 | | | 0.268 ± 0.041 | |
| [^177^Lu]Lu-PSMA-617 | 0.670 ± 0.031 | | 2.902 ± 0.164^*^ | | | 0.314 ± 0.083 | |
| [^177^Lu]Lu-PSMA-ALB-56 | 0.715 ± 0.071 | | 2.836 ± 0.313 | | | 0.254 ± 0.056 | |

^*^ Values significantly different from corresponding control group (*p*<0.05)

The albumin and blood urea nitrogen blood plasma levels, determined on Day 10 and 28 after injection of the PSMA radioligands, are described and discussed in the main article (Fig. 2 and Fig. 3) and listed in Table S2. Alkaline phosphatase levels decreased for all groups from Day 10 to Day 28, irrespective whether the mice had received radioligand therapy or only vehicle. Possibly, this observation can be ascribed to the age difference of the mice euthanized at these two timepoints. Total bilirubin levels in blood plasma were in the same range for all groups at both investigated timepoints.

**Table S2** Blood plasma parameters of mice determined on Day 10 and Day 28 after injection of 10 MBq radioligand. BUN = Blood urea nitrogen; ALP = alkaline phosphatase; TBIL = total bilirubin; ALB = albumin.

| **Day 10** | | | | |
| --- | --- | --- | --- | --- |
| **Group (n=4)** | **BUN** | **ALP** | **TBIL** | **ALB** |
|  | (mmol/L) | (U/L) | (µmol/L) | (g/L) |
| Vehicle | 7.05 ± 0.66 | 152 ± 28 | 3 ± 1 | 22 ± 1 |
| [^177^Lu]Lu-Ibu-DAB-PSMA | 9.23 ± 1.27^*^ | 122 ± 4 | 3 (n=2)  < 3 (n=2) | 23 ± 2 |
| [^177^Lu]Lu-PSMA-617 | 9.35 ± 0.88^*^ | 126 ± 29 | 3 (n=1)  < 3 (n=3) | 22 ± 2 |
| [^177^Lu]Lu-PSMA-ALB-56 | 8.88 ± 0.84^*^ | 135 ± 13 | 3 ± 1  < 3 (n=1) | 23 ± 3 |
| **Day 28** | | | | |
| **Group (n=4)** | **BUN** | **ALP** | **TBIL** | **ALB** |
|  | (mmol/L) | (U/L) | (µmol/L) | (g/L) |
| Vehicle | 7.27 ± 0.61 | 93 ± 11 | < 3 (n= 4) | 22 ± 1 |
| [^177^Lu]Lu-Ibu-DAB-PSMA | 9.02 ± 1.85 | 106 ± 3^*^ | 8 (n=1)  < 3 (n=3) | 23 ± 1 |
| [^177^Lu]Lu-PSMA-617 | 8.41 ± 0.81 | 102 ± 6 | 5 ± 3  < 3 (n=1) | 23 ± 1 |
| [^177^Lu]Lu-PSMA-ALB-56 | 9.69 ± 1.31^*^ | 96 ± 6 | < 3 (n=4) | 22 ± 1 |

^*^ Values significantly different from corresponding control group (*p*<0.05)

Histological investigations revealed no indications of acute toxicity to the kidneys, spleen and bone marrow of BALB/c nude mice after application of 10 MBq of the respective PSMA radioligands as no histological changes were observed in the tissues of treated mice as compared to the tissue of control mice (Fig. S2-S4).


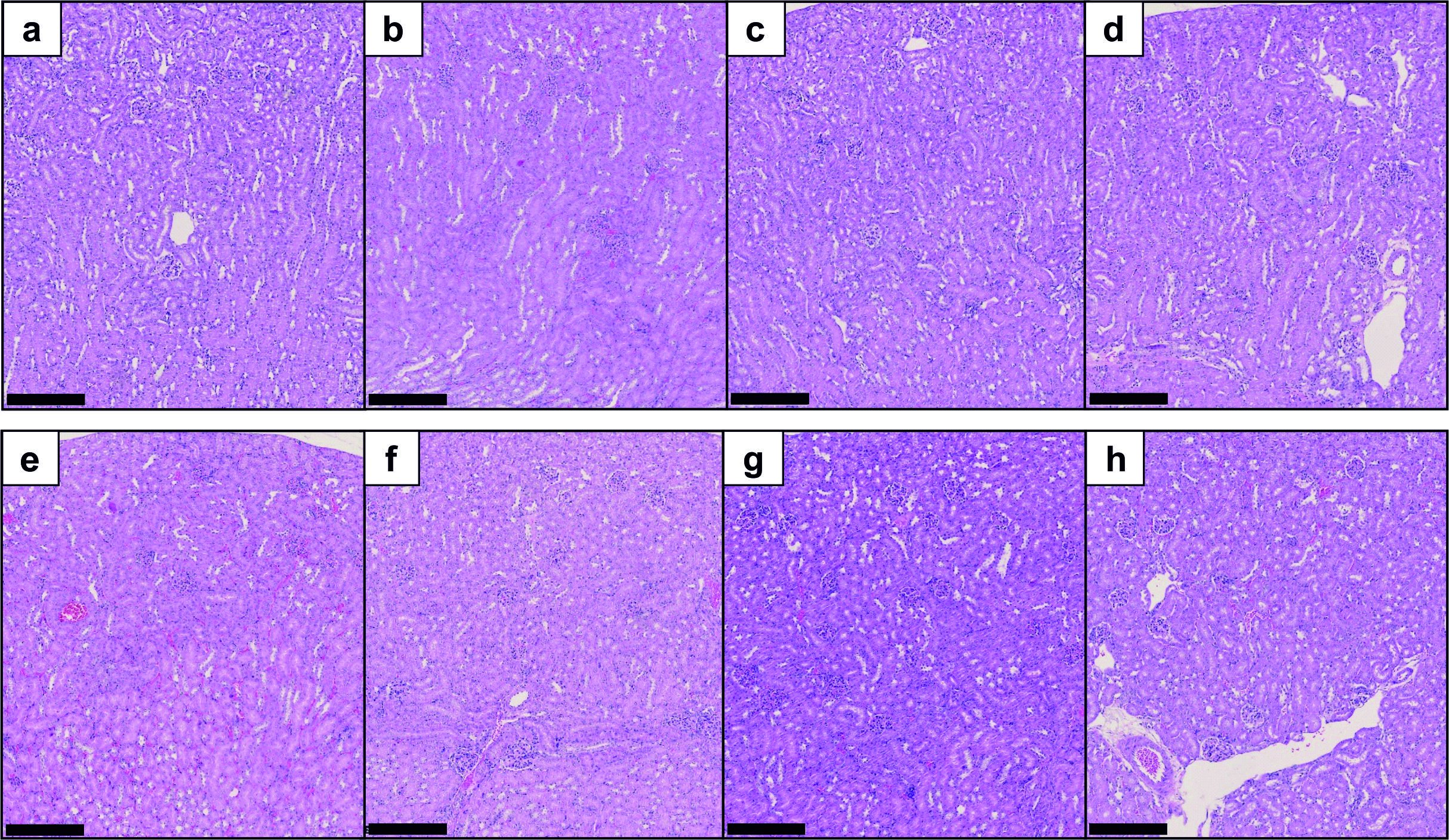


**Fig. S2** **a-h** Representative pictures of stained histological kidney sections of BALB/c nude mice. **a-d** Tissue collected on Day 10; **e-h** Tissue collected on Day 28 after administration of **a/e** vehicle (0.05% bovine serum albumin (BSA) in saline); **b/f** [^177^Lu]Lu-Ibu-DAB-PSMA (10 MBq per mouse); **c/g** [^177^Lu]Lu-PSMA-617 (10 MBq per mouse); **d/h** [^177^Lu]Lu-PSMA-ALB-56 (10 MBq per mouse). The black bar in the lower left corner of the images corresponds to 250 μm.


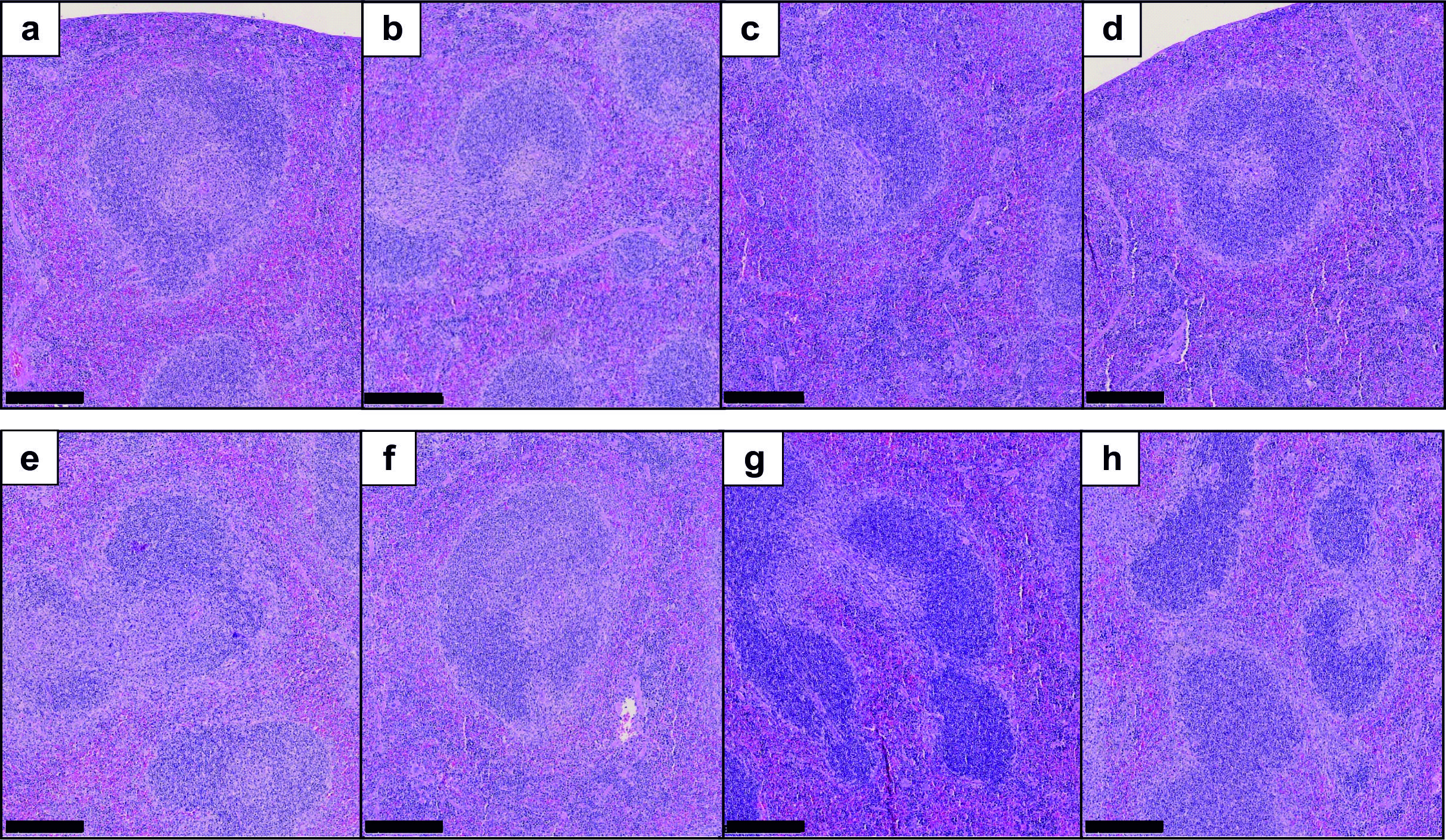


**Fig. S3 a-h** Representative pictures of stained histological sections of the spleen of BALB/c nude mice. **a-d** Tissue collected on Day 10; **e-h** Tissue collected on Day 28 after administration of **a/e** vehicle (0.05% BSA in saline); **b/f** [^177^Lu]Lu-Ibu-DAB-PSMA (10 MBq per mouse); **c/g** [^177^Lu]Lu-PSMA-617 (10 MBq per mouse); **d/h** [^177^Lu]Lu-PSMA-ALB-56 (10 MBq per mouse). The black bar in the lower left corner of the images corresponds to 250 μm.


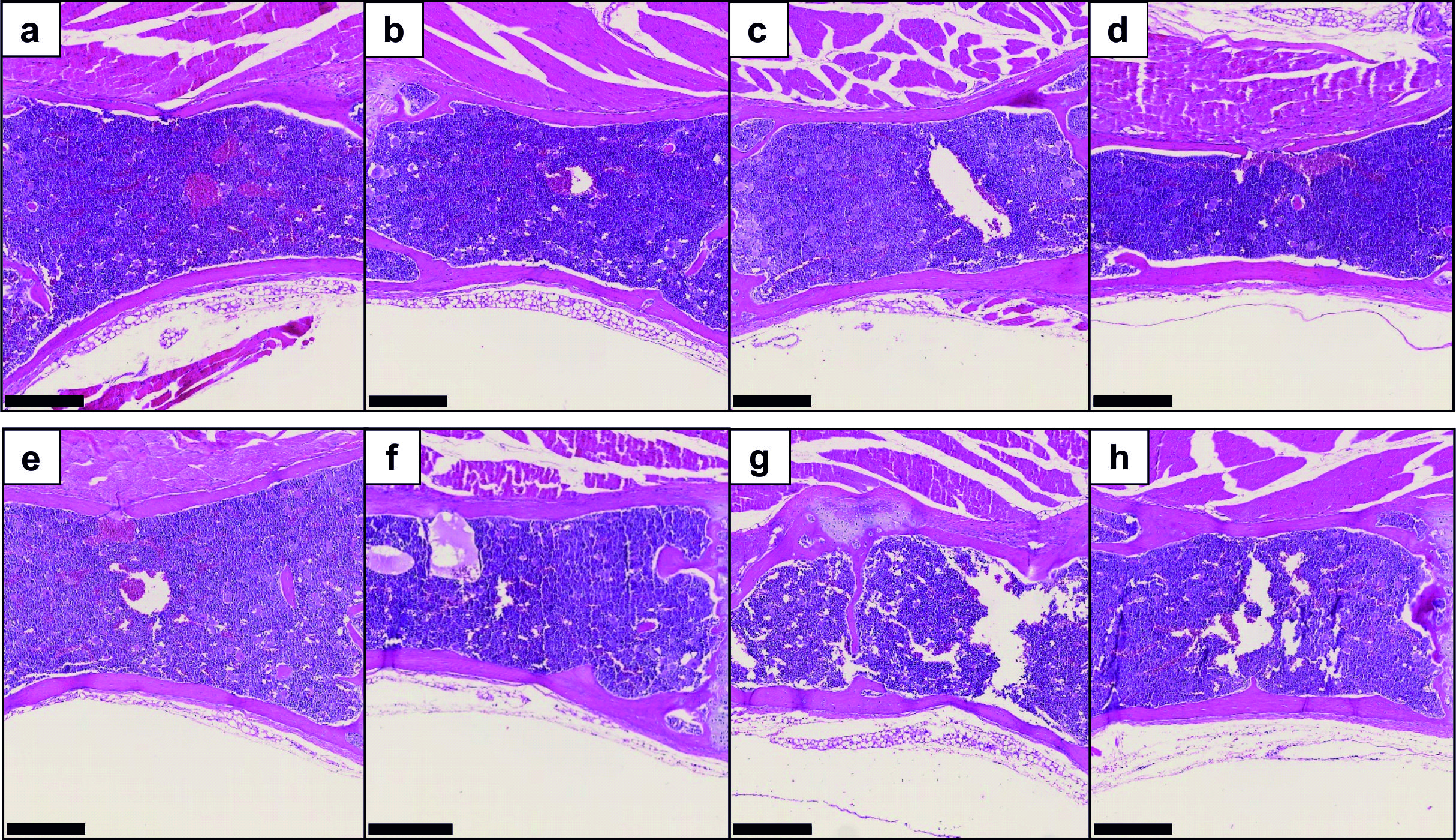


**Fig. S4** **a-h** Representative pictures of stained histological sections of the bone marrow of BALB/c nude mice. **a-d** Tissue collected on Day 10; **e-h** Tissue collected on Day 28 after administration of **a/e** vehicle (0.05% BSA in saline); **b/f** [^177^Lu]Lu-Ibu-DAB-PSMA (10 MBq per mouse); **c/f** [^177^Lu]Lu-PSMA-617 (10 MBq per mouse); **d/h** [^177^Lu]Lu-PSMA-ALB-56 (10 MBq per mouse). The black bar in the lower left corner of the images corresponds to 250 μm.

**4. Body mass of tumor-bearing mice during the therapy study**

**Purpose:** As a means of monitoring radioligand therapy (RLT), the absolute and relative body masses of mice were determined as they can be considered as a measure of the general health condition of mice.

**Methods:** During RLT, the body mass of each mouse was measured every second day. The relative body mass was calculated based on the formula [body mass(x)/body mass(0)], in which the body mass(x) is the body mass in grams on a given Day x and body mass(0) is the body mass in grams on Day 0.

**Results:** The body mass of all groups of mice treated with 2 MBq of any of the radioligands or only vehicle decreased over the course of the study. This observation can be ascribed to the fast tumor growth, which affected the overall condition of the mice, resulting in significantly lower body masses at the time of euthanasia as compared to the body masses of other groups (*p*<0.05) (Fig. S5a/b). The same situation held true for mice treated with 5 MBq [^177^Lu]Lu-PSMA-617, which did not effectively reduce tumor growth (Fig. S5c/d). Mice treated with 10 MBq [^177^Lu]Lu-PSMA-617, 5 MBq or 10 MBq [^177^Lu]Lu-Ibu-DAB-PSMA as well as mice treated with 5 MBq or 10 MBq [^177^Lu]Lu-PSMA-ALB-56 showed an increase in body mass over the course of the therapy study as would be the case for mice without tumors (Fig. S5c-f).


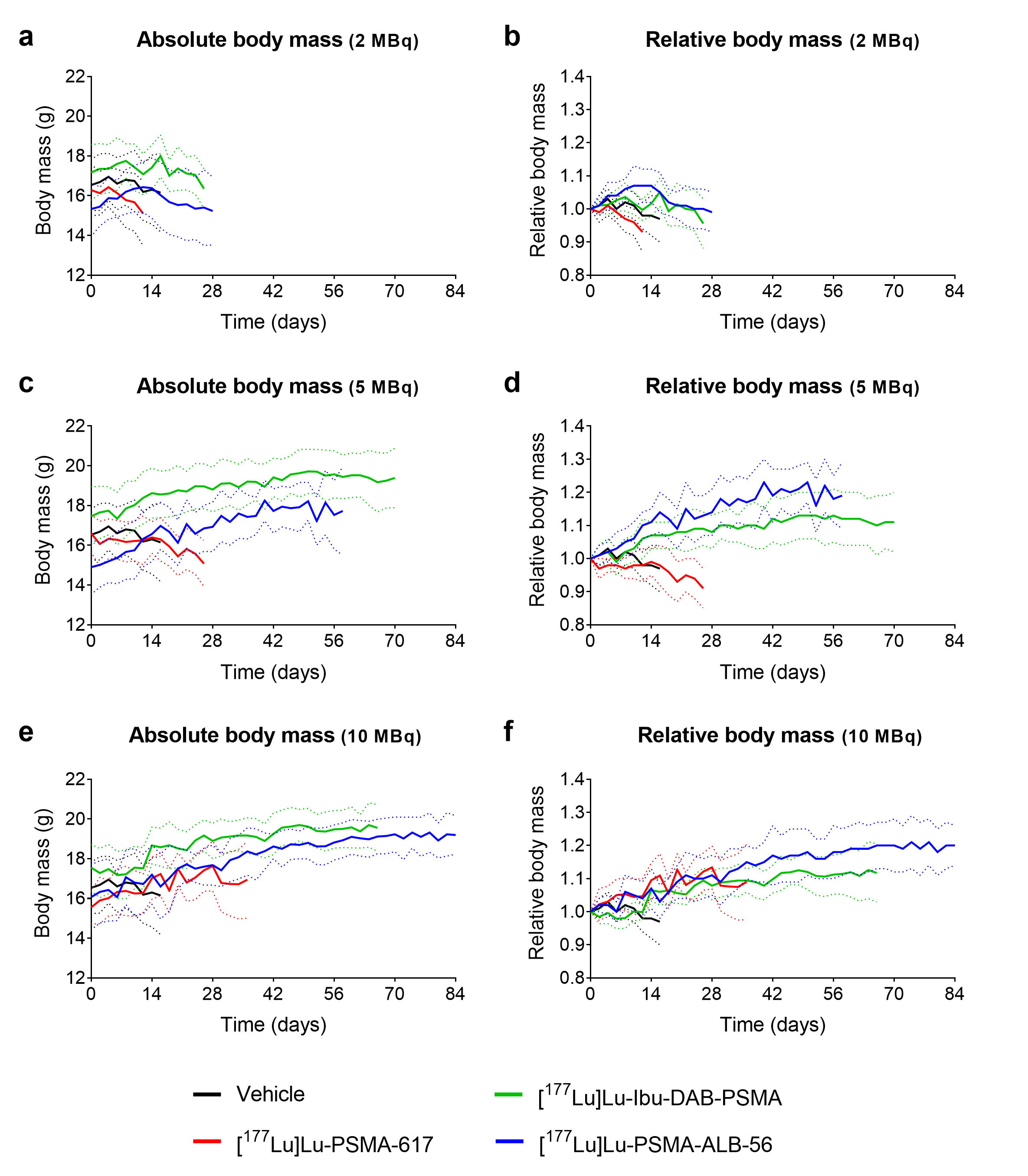


**Fig. S5 a-f** Body masses of mice over the time course of the therapy study presented as **(a/c/e)** absolute body masses and **(b/d/f)** relative body masses. Data are shown until the first mouse of each group reached an endpoint and are presented as the average of treated mice (n=6-12) or untreated controls (n=21) with dashed lines indicating the ± SD. The absolute and relative body mass of mice injected with vehicle are based on data of mice in the control group combined with values of control mice from a previous study published by Umbricht et al. Mol Pharm 2018; 15:2297−2306. Copyright 2022 American Chemical Society [4]. Curves of mice injected with [^177^Lu]Lu-PSMA-617 (2 MBq and 5 MBq) and [^177^Lu]Lu-PSMA-ALB-56 (2 MBq and 5 MBq) are based on previously obtained data for comparison, published in the same article published by Umbricht et al. [4].

**5. Assessment of potential impairment of blood cells in immunocompetent mice**

**Purpose:** Potential differences among the radioligands’ effects on hematological parameters were investigated in normal, immunocompetent (FVB) mice.

**Methods:** Immunocompetent FVB mice (n=4 per group) were intravenously injected with 30 MBq (1 nmol) of [^177^Lu]Lu-Ibu-DAB-PSMA, [^177^Lu]Lu-PSMA-617 or [^177^Lu]Lu-PSMA-ALB-56. Three times a week, the body mass of the mice was measured and the animals were assessed for signs of pain or unease. On Day 10 and Day 28, blood was taken from the sublingual vein and collected in EDTA tubes (Microvette® 100 K3 EDTA, Sarstedt, Germany) for analysis of the hemograms and the preparation of blood smears.

*Hemogram/full blood counts:* Erythrocyte, leukocyte, lymphocyte and thrombocyte counts as well as hemoglobin concentration and the hematocrit were determined using a hematology analyzer (VetScan HM5, Abaxis, United States).

*Pappenheim staining method of blood cell smears:* Blood smears were prepared on microscopy slides. After fixation in methanol, the slides were stained in a May Grünwald solution and subsequently in a diluted Giemsa solution [7]. The slides were rinsed with Milli-Q water, dried and covered with mounting medium (Pertex) using coverslips. Potential abnormalities in the morphology and number of blood cells were assessed by a veterinary clinical pathologist.

**Results:** The results of the hemograms are reported in the main article (Fig. 5). Blood smears obtained on Day 10 and Day 28 after the application of 30 MBq PSMA radioligand were analyzed with regard to the percentage of lymphocytes, neutrophils, monocytes, eosinophils and basophils based on the total leukocyte counts (set as 100%) and compared to the control group (Table S3). Mice that received [^177^Lu]Lu-PSMA-617 showed a higher percentage of lymphocytes (93 ± 3%) on Day 10 compared to untreated control mice (86 ± 3%). No further significant differences between the treated groups and the control group were observed at this timepoint. On Day 28, the mice that received [^177^Lu]Lu-PSMA-ALB-56 showed a significantly lower percentage of lymphocytes (76 ± 5%) compared to untreated controls (90 ± 4%). Moreover, the percentage of neutrophils was increased (19 ± 7%) in this group as compared to control values (8.0 ± 3.7%) obtained at the same timepoint (Table S3). Basophils were not listed as their values were commonly below 1% in agreement with the literature [8].

**Table S3** Blood smear-based analysis of leukocyte subgroups determined on Day 10 and on Day 28 after injection of PSMA radioligand (30 MBq/mouse) compared to the values of control mice. The data are indicated as percentage of leukocytes ± SD.

| **Day 10** | | | | |
| --- | --- | --- | --- | --- |
| **Group (n=4)** | **Lymphocytes**  **(%)** | **Neutrophils**  **(%)** | **Monocytes**  **(%)** | **Eosinophils**  **(%)** |
| Control | 86 ± 3 | 9.5 ± 4.4 | 3.5 ± 2.5 | 1.5 ± 1.0 |
| [^177^Lu]Lu-Ibu-DAB-PSMA | 92 ± 4 | 6.5 ± 3.0 | 1.0 ± 1.2 | 0.5 ± 1.0 |
| [^177^Lu]Lu-PSMA-617 | 93 ± 3* | 5.0 ± 3.5 | 2.0 | 0 |
| [^177^Lu]Lu-PSMA-ALB-56 | 82 ± 4 | 12 ± 4 | 4.0 ± 2.3 | 2.5 ± 2.5 |
| **Day 28** | | | | |
| **Group (n=4)** | **Lymphocytes**  **(%)** | **Neutrophils**  **(%)** | **Monocytes**  **(%)** | **Eosinophils**  **(%)** |
| Control | 90 ± 4 | 8.0 ± 3.7 | 2.0 ± 1.6 | 0 |
| [^177^Lu]Lu-Ibu-DAB-PSMA | 89 ± 5 | 7.0 ± 5.8 | 4.0 ± 1.6 | 0 |
| [^177^Lu]Lu-PSMA-617 | 88 ± 3 | 7.0 ± 3.5 | 5.0 ± 2.0 | 0 |
| [^177^Lu]Lu-PSMA-ALB-56 | 76 ± 5* | 19 ± 7* | 5.0 ± 3.8 | 1.0 ± 1.2 |

^*^ Values significantly different from corresponding control group (*p*<0.05)

Basophil values were commonly < 1% and, therefore, not explicitly listed.

The body masses of mice in all groups that were injected with 30 MBq [^177^Lu]Lu-Ibu-DAB-PSMA, [^177^Lu]Lu-PSMA-617 or [^177^Lu]Lu-PSMA-ALB-56 were in the same range and did not significantly differ from the body mass of mice in the control group at treatment start and on Day 10 and Day 28 p.i. (Fig. S6).

**
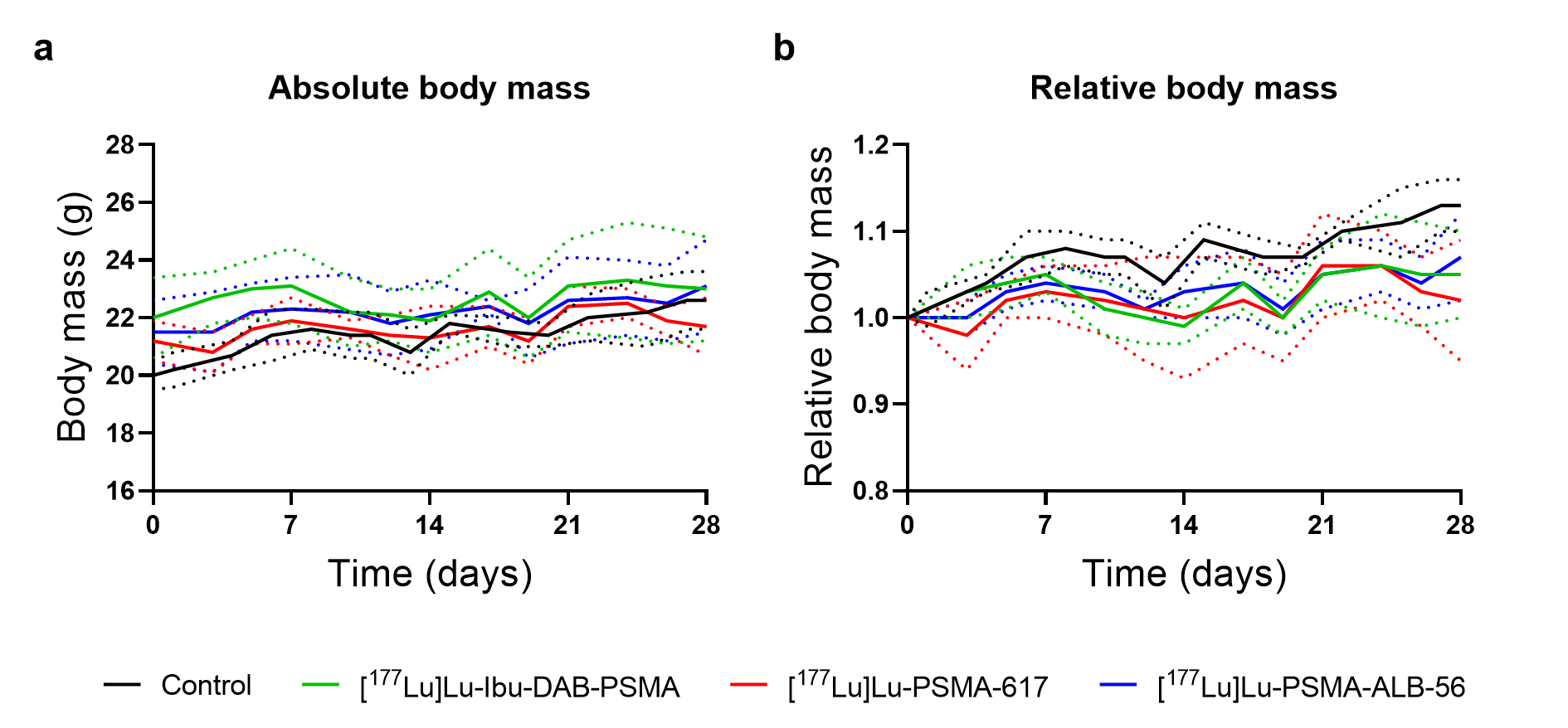
**

**Fig. S6 a/b** Body masses of FVB mice over the time course of the tolerability study presented as **(a)** absolute body masses and **(b)** relative body masses. Data are shown until the first mouse of each group reached an endpoint and are presented as the average of treated mice or untreated controls (n=4) with dashed lines indicating the ± SD.

**6. Comparison of the blood AUC_0→192h_ values of the radioligands**

**Purpose:** The area under the curve values (AUC_0→192h_) for the blood were determined for [^177^Lu]Lu-Ibu-DAB-PSMA, [^177^Lu]Lu-PSMA-617 and [^177^Lu]Lu-PSMA-ALB-56 in order to compare their blood retention and estimate the differences in absorbed bone marrow dose.

**Methods:** The areas under the curve (AUC_0→192h_) were calculated for the blood based on non-decay-corrected time-dependent biodistribution data previously obtained in PC-3 PIP tumor-bearing mice [2-4] using GraphPad Prism software (version 8). The radioligand uptake in the blood immediately after injection (t = 0) was set as 100% injected activity (IA)/g based on the assumption that the blood volume of a mouse was ~1 mL (i.e. 1.0 g), which means about 6% of the body mass which was 17–18 g [9].

**Results:** The blood AUC_0→192h_ value of [^177^Lu]Lu-Ibu-DAB-PSMA was 2.6-fold lower than the value for [^177^Lu]Lu-PSMA-ALB-56 but 2.5-fold higher than for [^177^Lu]Lu-PSMA-617. This means that the blood AUC_0→192h_ was 6.6-fold higher for [^177^Lu]Lu-PSMA-ALB-56 than for [^177^Lu]Lu-PSMA-617 (Fig. S7, Table S4).


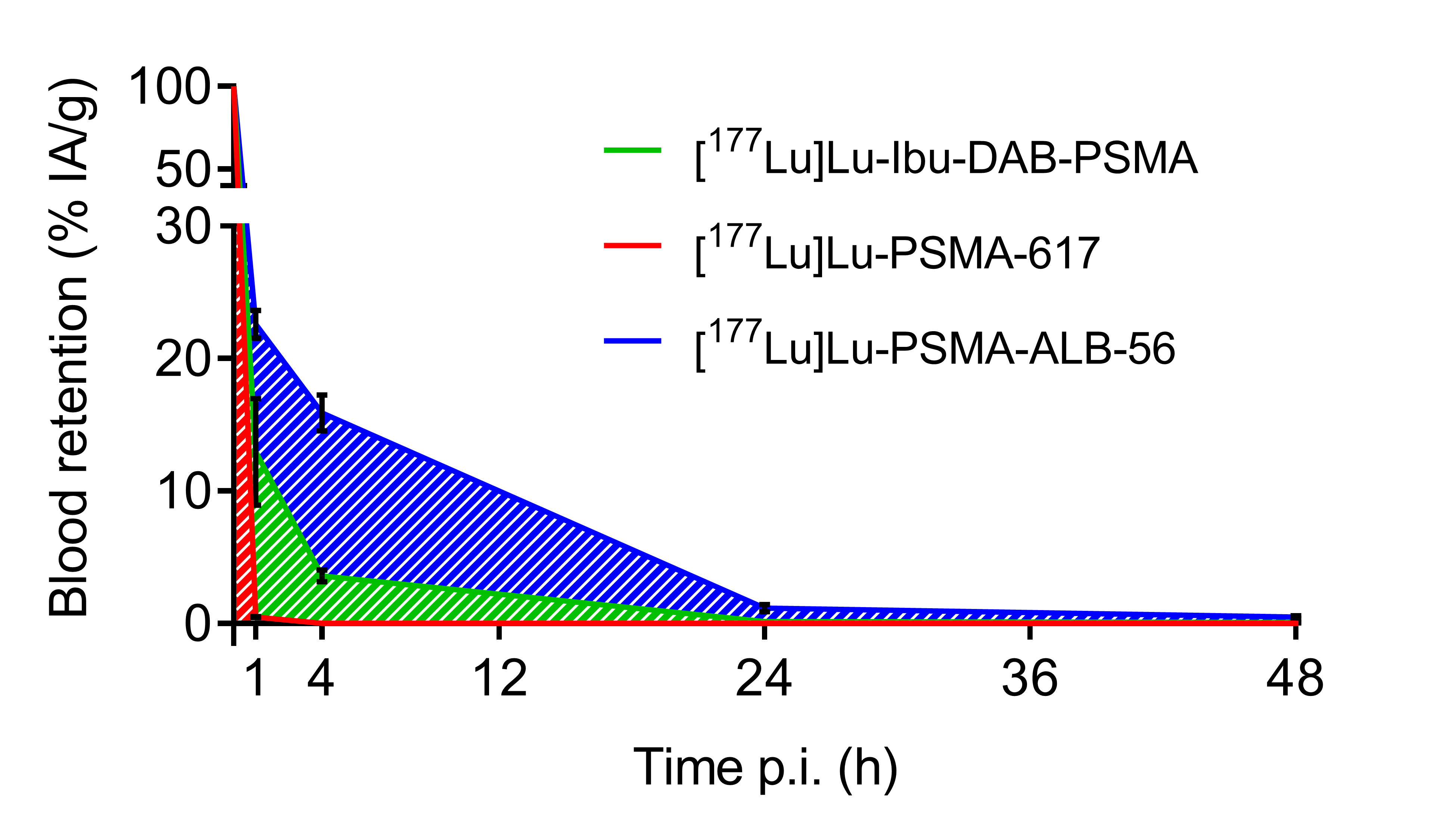


**Fig. S7** Visualization of the areas under the curve for the blood retention of the different PSMA-binding radioligands over the first 48 h after injection. The data points are based on previously reported biodistribution results of [^177^Lu]Lu-Ibu-DAB-PSMA (Deberle & Benešová et al. Theranostics 2020; 10:1678−1693 [2]), [^177^Lu]Lu-PSMA-617 (Benešová et al. Mol Pharm 2018;15:934−946. Copyright 2022 American Chemical Society [3]) and [^177^Lu]Lu-PSMA-ALB-56 (Umbricht et al. Mol Pharm 2018; 15:2297−2306. Copyright 2022 American Chemical Society [4]). The curves are shown up to 48 h post injection (p.i) when the activity was almost completely cleared. Each data point represents the average of a group of n=3−6 mice ± SD indicated as non-decay corrected percentage of injected activity per gram blood [% IA/g].

**Table S4** Areas under the curve (AUC_0→192h_) indicated as [% IA/g‧h]-values for the blood. The values are indicated as average ± standard error (SE) based on non-decay-corrected biodistribution data

|  | **[^177^Lu]Lu-Ibu-DAB-PSMA^a^** | **[^177^Lu]Lu-PSMA-617^b^** | **[^177^Lu]Lu-PSMA-ALB-56^c^** |
| --- | --- | --- | --- |
| Blood AUC_0→192h_ | 129 ± 8 | 52 ± 2 | 341 ± 17 |
| Relative values | 2.5 | 1.0 | 6.6 |

^a^Biodistribution data of [^177^Lu]Lu-Ibu-DAB-PSMA were previously published by Deberle & Benešová et al. 2020 [2]; ^b^Biodistribution data of [^177^Lu]Lu-PSMA-617 were previously published by Benešová et al. 2018 [3]; ^c^Biodistribution data of [^177^Lu]Lu-PSMA-ALB-56 were previously published by Umbricht et al. 2018 [4].

**References**

1. Salvat F. PENELOPE2014: A code system for Monte-Carlo simulation of electron and photon transport. OECD/NEA Data Bank: NEA/NSC/DOC. 2015;3.

2. Deberle LM, Benesova M, Umbricht CA, Borgna F, Büchler M, Zhernosekov K, et al. Development of a new class of PSMA radioligands comprising ibuprofen as an albumin-binding entity. Theranostics. 2020;10:1678-93. doi:10.7150/thno.40482.

3. Benešová M, Umbricht CA, Schibli R, Müller C. Albumin-binding PSMA ligands: optimization of the tissue distribution profile. Mol Pharm. 2018;15:934-46. doi:10.1021/acs.molpharmaceut.7b00877.

4. Umbricht CA, Benešová M, Schibli R, Müller C. Preclinical development of novel PSMA-targeting radioligands: modulation of albumin-binding properties to improve prostate cancer therapy. Mol Pharm. 2018;15:2297-306. doi:10.1021/acs.molpharmaceut.8b00152.

5. Siwowska K, Guzik P, Domnanich KA, Monne Rodriguez JM, Bernhardt P, Ponsard B, et al. Therapeutic potential of ^47^Sc in comparison to ^177^Lu and ^90^Y: preclinical investigations. Pharmaceutics. 2019;11. doi:10.3390/pharmaceutics11080424.

6. Haller S, Pellegrini G, Vermeulen C, van der Meulen NP, Köster U, Bernhardt P, et al. Contribution of Auger/conversion electrons to renal side effects after radionuclide therapy: preclinical comparison of ^161^Tb-folate and ^177^Lu-folate. EJNMMI Res. 2016;6:13. doi:10.1186/s13550-016-0171-1.

7. Binder T, Diem H, Fuchs R, Gutensohn K, Nebe T. Pappenheim stain: description of a hematological standard stain - history, chemistry, procedure, artifacts and problem solutions. Journal of Laboratory Medicine. 2012;36:293-309. doi:10.1515/labmed-2012-0027.

8. Schneck K, Washington M, Holder D, Lodge K, Motzel S. Hematologic and serum biochemical reference values in nontransgenic FVB mice. Comp Med. 2000;50:32-5.

9. Raabe BM, Artwohl JE, Purcell JE, Lovaglio J, Fortman JD. Effects of weekly blood collection in C57BL/6 mice. J Am Assoc Lab Anim Sci. 2011;50:680-5.
